# Automated Segmentation of the Mouse Body Language to Study Stimulus-Evoked Emotional Behaviors

**DOI:** 10.1523/ENEURO.0514-22.2023

**Published:** 2023-09-08

**Authors:** Gabriele Chelini, Enrico Maria Trombetta, Tommaso Fortunato-Asquini, Ottavia Ollari, Tommaso Pecchia, Yuri Bozzi

**Affiliations:** 1 Center for Mind/Brain Sciences (CIMeC), University of Trento, Rovereto 38068, Italy; 2 Department of Cellular, Computational, and Integrative Biology (CIBIO), University of Trento, Trento 38123, Italy; 3 Consiglio Nazionale delle Ricerche (National Council of Research) Neuroscience Institute, Pisa 56124, Italy

**Keywords:** behavioral segmentation, DeepLabCut, mouse behavior, mouse body language, mouse emotion, whisker nuisance

## Abstract

Understanding the neural basis of emotions is a critical step to uncover the biological substrates of neuropsychiatric disorders. To study this aspect in freely behaving mice, neuroscientists have relied on the observation of ethologically relevant bodily cues to infer the affective content of the subject, both in neutral conditions or in response to a stimulus. The best example of that is the widespread assessment of freezing in experiments testing both conditioned and unconditioned fear responses. While robust and powerful, these approaches come at a cost: they are usually confined within selected time windows, accounting for only a limited portion of the complexity of emotional fluctuation. Moreover, they often rely on visual inspection and subjective judgment, resulting in inconsistency across experiments and questionable result interpretations. To overcome these limitations, novel tools are arising, fostering a new avenue in the study of the mouse naturalistic behavior. In this work we developed a computational tool [stimulus-evoked behavioral tracking in 3D for rodents (SEB3R)] to automate and standardize an ethologically driven observation of freely moving mice. Using a combination of machine learning-based behavioral tracking and unsupervised cluster analysis, we identified statistically meaningful postures that could be used for empirical inference on a subsecond scale. We validated the efficacy of this tool in a stimulus-driven test, the whisker nuisance (WN) task, where mice are challenged with a prolonged and invasive whisker stimulation, showing that identified postures can be reliably used as a proxy for stimulus-driven fearful and explorative behaviors.

## Visual Abstract

**Figure:**
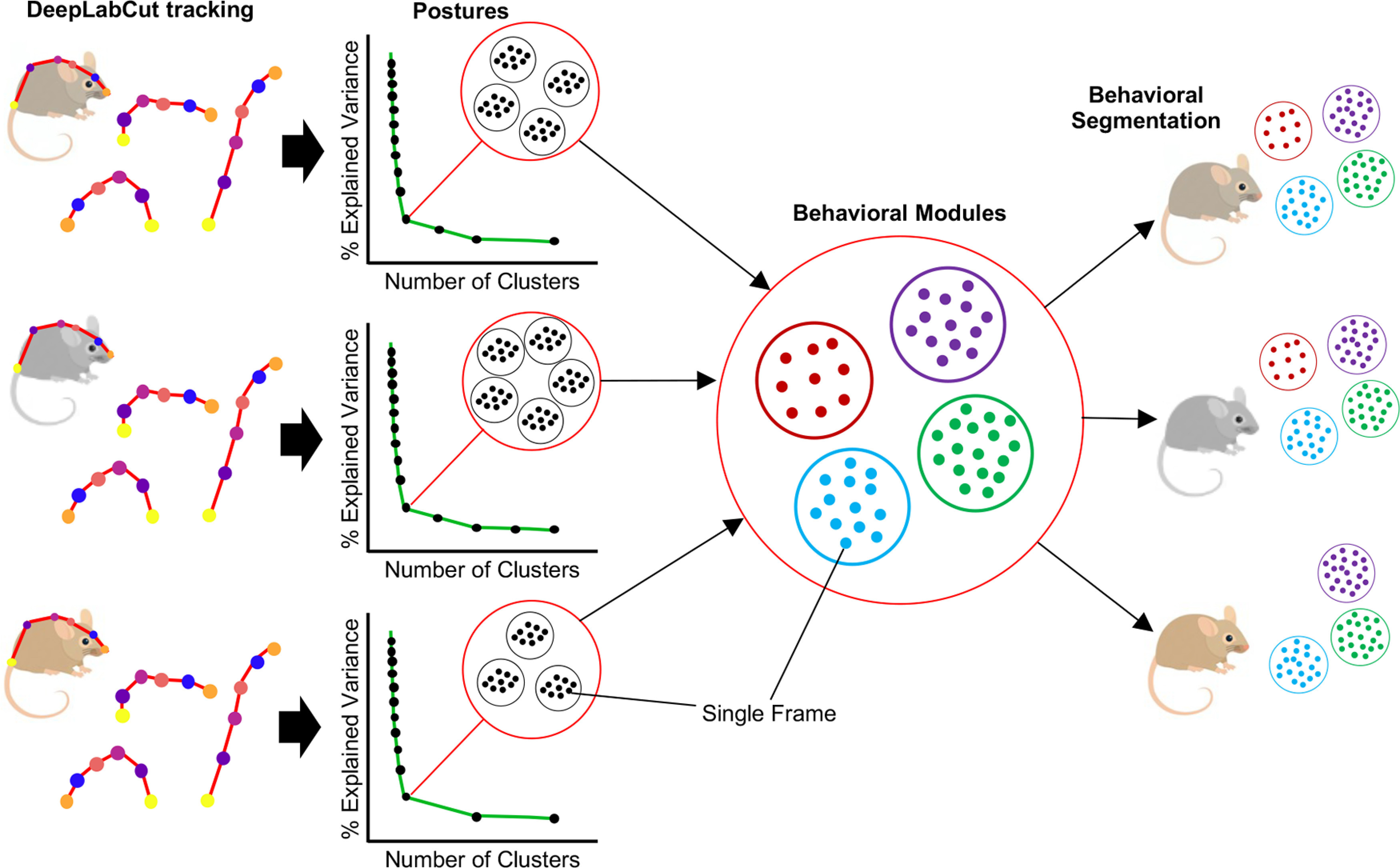

## Significance Statement

We combined novel technical advancements of system neuroscience with a traditional ethology-based behavioral observation to design a simple computational tool for behavior detection in freely moving mice. Using machine learning-based behavioral tracking and unsupervised cluster analysis, we identified statistically meaningful postures on a subsecond scale. We validated this tool in the whisker nuisance (WN) task, where mice are challenged with a prolonged and invasive whisker stimulation, showing that identified postures can be used as a proxy for stimulus-driven fearful and explorative behaviors. With this tool we aim to automate, accelerate and standardize data collection across research laboratories, improving the quality and reproducibility of behavioral studies in mice.

## Introduction

**Figure 1.**
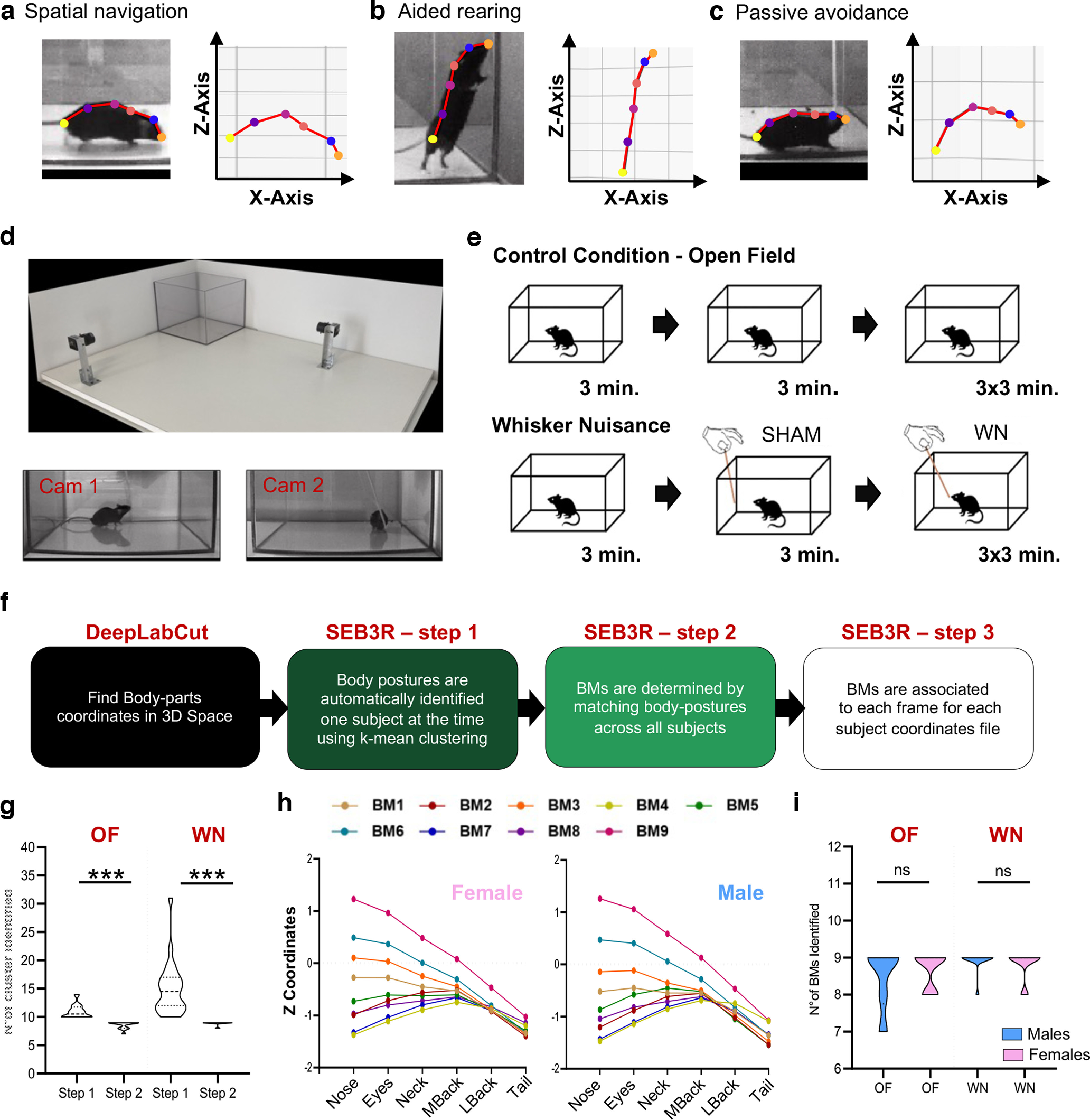
SEB3R pipeline. ***a–c***, Examples of body postures determined by visual inspection (left) and corresponding coordinates detected by DLC-3D. Bodily hotspots are indicated by colored dots (orange, nose; blue, eyes; pale red, neck; magenta, mid back; purple, lower back; yellow, tail attachment). ***d***, Top, Angular view of the arena for 3D motion capture. Bottom, Orthogonal view of the same mouse during the WN. ***e***, Design of the validation experiment. Top, Open field (OF). Bottom, Whisker nuisance task. ***f***, Schematic workflow of the SEB3R pipeline. ***g***, A two-step cluster analysis eliminates interindividual variability. ***h***, Schematic view of BMs identified during the open field session, for both sexes. ***i***, The final number of BMs is insensitive to size-biases, thus resulting in analogous identification in both sexes (Extended Data Figs. 1-1, 1-2, 1-3, 1-4, 1-5, 1-6 show screengrabs for the critical outputs of the SEB3R pipeline). ***p < 0.0001; ns, not significant.

As with any behaving animal, naturalistic mouse behavior incorporates innate strategies to balance explorative and aversive responses to the surrounding environment. Neuroscientists often exploit this dichotomy to design behavioral tests assessing mouse emotional behavior. Many of these tests put the animal in a forced choice between a putatively safe location versus a potentially threatening alternative (Takao and Miyakawa, 2006; Komada et al., 2008; Bailey and Crawley, 2009; Campos et al., 2013; Kraeuter et al., 2019). Other approaches use stimulus-driven tasks to assess the positive or negative valence of the emotional response to a reward or fearful cue respectively, using one specific behavioral output as a proxy to infer emotional state (such as self-administration of a positive stimulus or freezing in response to a threat; Panlilio and Goldberg, 2007; Campos et al., 2013; Gafford and Ressler, 2016). While extremely valuable, none of these approaches address the dynamic change in the subjects’ affective state over extended periods, limiting results interpretation to circumscribed context rather than interrogating the emotional fluctuation occurring during ecological behavioral flow. Thanks to the advent of advanced tools for behavioral tracking and unbiased computer-driven classification, novel ethologically relevant methods to classify mouse emotions are arising (Wiltschko et al., 2015; Mathis et al., 2018; Nath et al., 2019; Dolensek et al., 2020; Bohnslav et al., 2021; Hsu and Yttri, 2021; Karashchuk et al., 2021; Luxem et al., 2022), promising a new era in the use of mouse behavior as an optimal translational tool for neuropsychiatric research. To fully exploit the potential of these methods, the field craves for open-access resources to break-down and analyze the output. For instance, using the pose-estimation software DeepLabCut (DLC), it is possible to reliably track several bodily hotspots on a behaving animal, allowing for virtually limitless identification of animal behavioral components. However, every new tracking configuration will require dedicated analysis pipeline to swiftly transform DLC output files into interpretable results. Generate and sharing tools to perform this transformation is an integral step to standardize behavioral assessment across research laboratories, thus improving interpretation and replicability. In this work, we conceptualized an automated procedure to discriminate mouse body postures on a subsecond scale that we named SEB3R (stimulus-evoked behavioral tracking in 3D for rodents), using a combination of machine-learning based behavioral tracking and unsupervised cluster analysis. After visualizing ∼100 videos of freely moving mice responding to a nonpainful whisker stimulation, we concluded that a good predictor of mice affective response could be simplified as the spatial location, along the vertical (*z*)-axis, of specific “bodily hotspots”: nose, eyes, neck, mid back, lower back, tail attachment (Fig. 1*a–c*). More specifically, the relative position of one hotspot compared with the others changes drastically according to specific behavioral states such as spatial navigation, explorative rearing, or passive avoidance (Fig. 1*a–c*). Taking advantage of the new-generation software for pose estimation DeepLabCut (DLC; Mathis et al., 2018; Nath et al., 2019), we tracked these hotspots in freely moving mice and used the output coordinates to read mice body language. To validate this method, we compared behavior in mice navigating undisturbed an open field (OF) arena against a group of littermates challenged with an invasive whisker stimulation [whisker nuisance task (WN); McNamara et al., 2010; Fontes-Dutra et al., 2018; Chelini et al., 2019; Balasco et al., 2022a; Fig. 1*e*].

10.1523/ENEURO.0514-22.2023.f1-1 Extended Data Figure 1-1 Example of the output file MouseIDDistMean.CSV provided at the end of step 1 and required to run step 2 for one representative mouse (animal number 156). Download Figure 1-1, TIF file.

**Figure 2.**
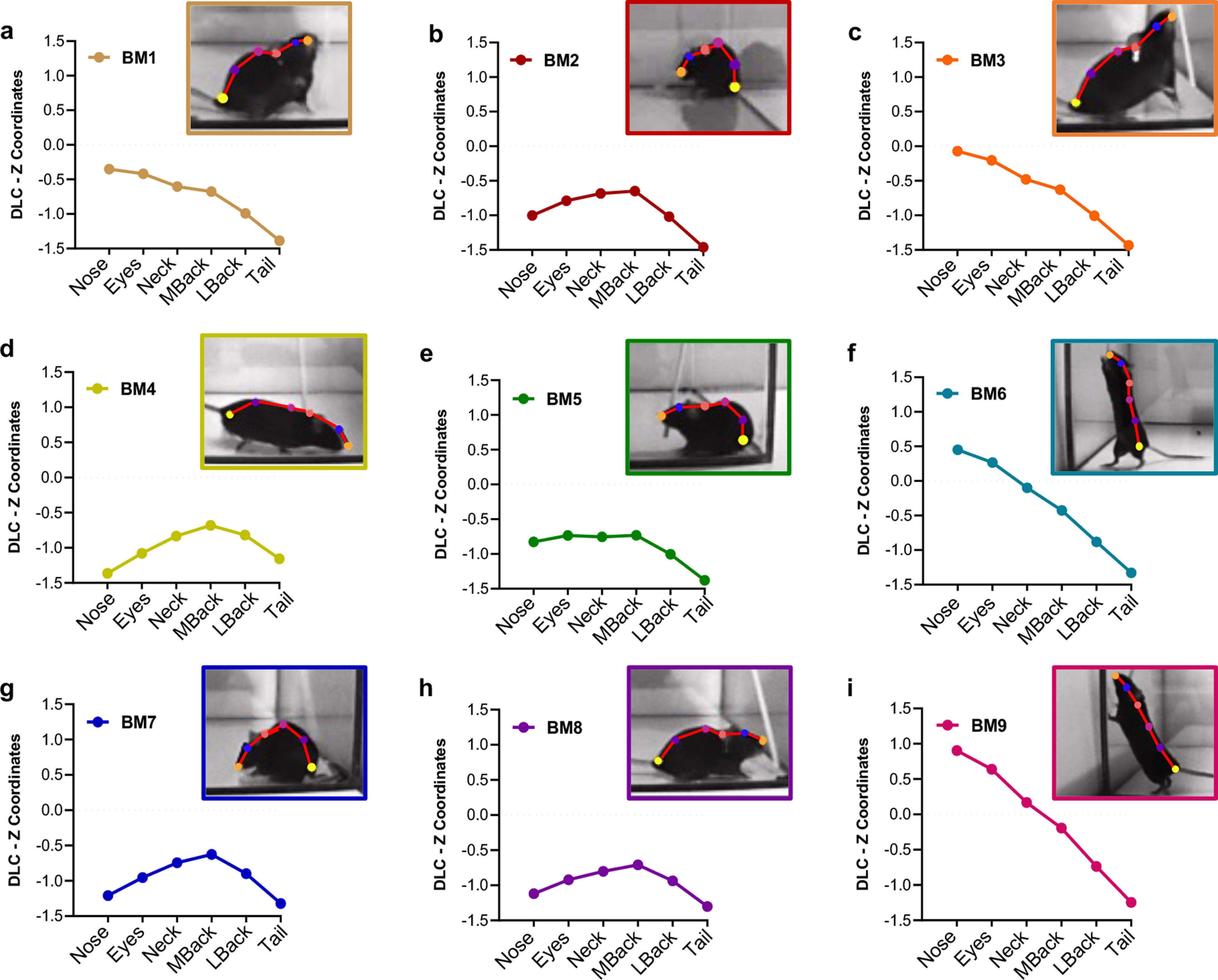
Behavioral modules (BMs) identified by SEB3R. ***a–i***, The plots report the DLC *z*-coordinates for each of the six bodily hotspots (nose, eyes, neck, mid back, lower back, and tail attachment) that allow to identify each of the nine BMs. A representative frame is reported for each BM.

10.1523/ENEURO.0514-22.2023.f1-2 Extended Data Figure 1-2 Pop-up window asking the user to indicate the final number of *k-means* required to run the step 2. The indicated *k-mean* will correspond to the final number of BMs. Download Figure 1-2, TIF file.

**Figure 3.**
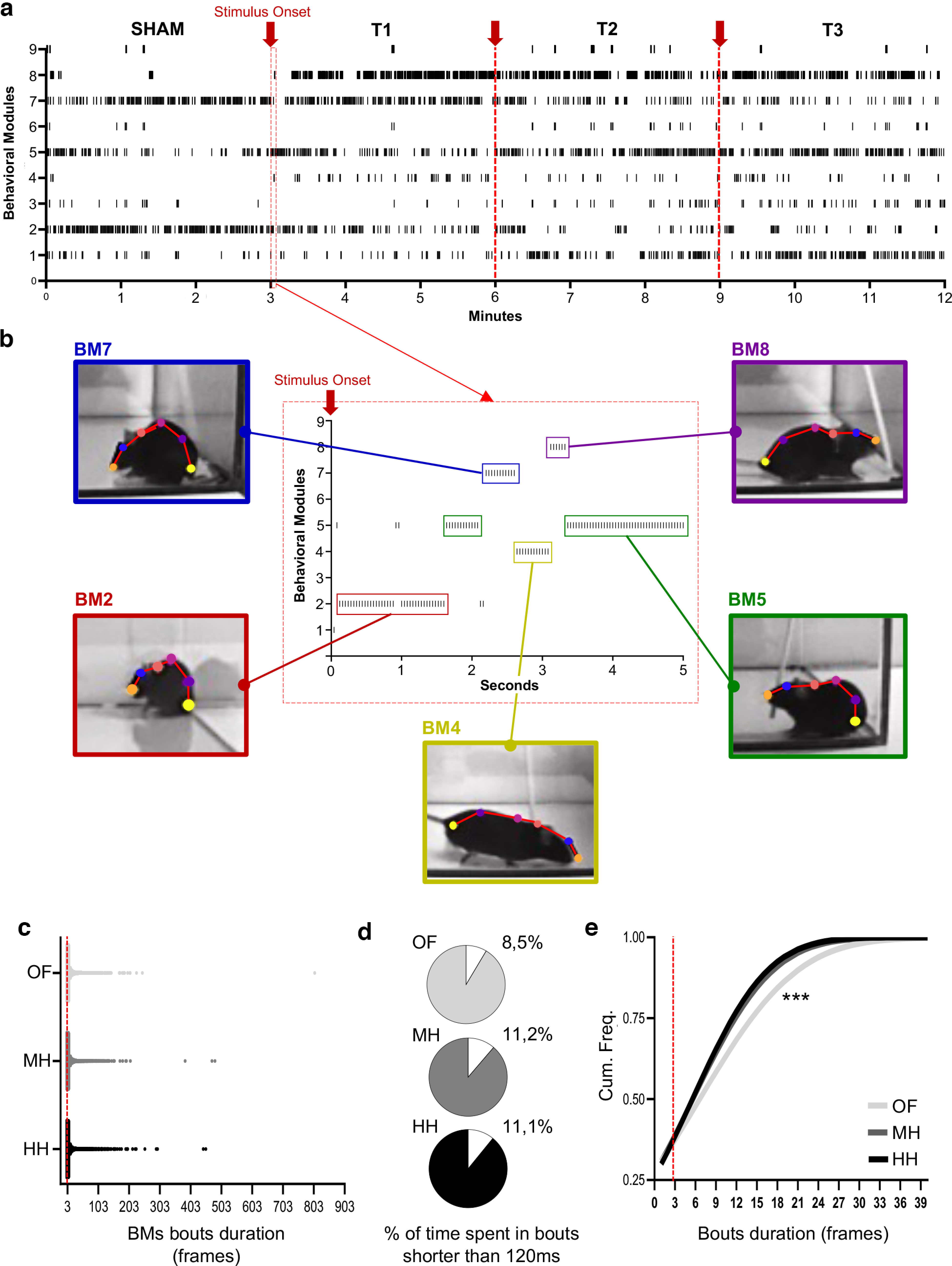
Behavioral recognition on a frame-by-frame resolution in the WN. ***a***, Raster plot showing the occurrence of BMs in a prototypical mouse for the entire duration of the WN. Each vertical bar (**|**) indicates a BM detected in the corresponding period (sham session and trial sessions T1–T3). Stimulus onset at the beginning of each stimulation trial is indicated by a red arrow. ***b***, Example of BMs detected during the first 5 s of whisker stimulation in the WN; the *x*-axis reports the seconds after the beginning of whisker stimulation in T1. ***c***, Violin plot showing the spread in the duration of BMs sequences. ***d***, Percentage of frames belonging to BMs sequences shorter than three frames (120 ms) is lower in the OF condition compared with WN. ***e***, Cumulative frequencies chart shows that BMs sequences are longer in in the OF compared with WN (*x*-axis is cropped at a duration of 39 frames to better appreciate the differences; all 3 curves perfectly overlap after this point). ***p < 0.0001.

10.1523/ENEURO.0514-22.2023.f1-3 Extended Data Figure 1-3 Example of the output file ModulesAssigned.CSV provided at the end of step 2 and required to run step 3. Download Figure 1-3, TIF file.

**Figure 4.**
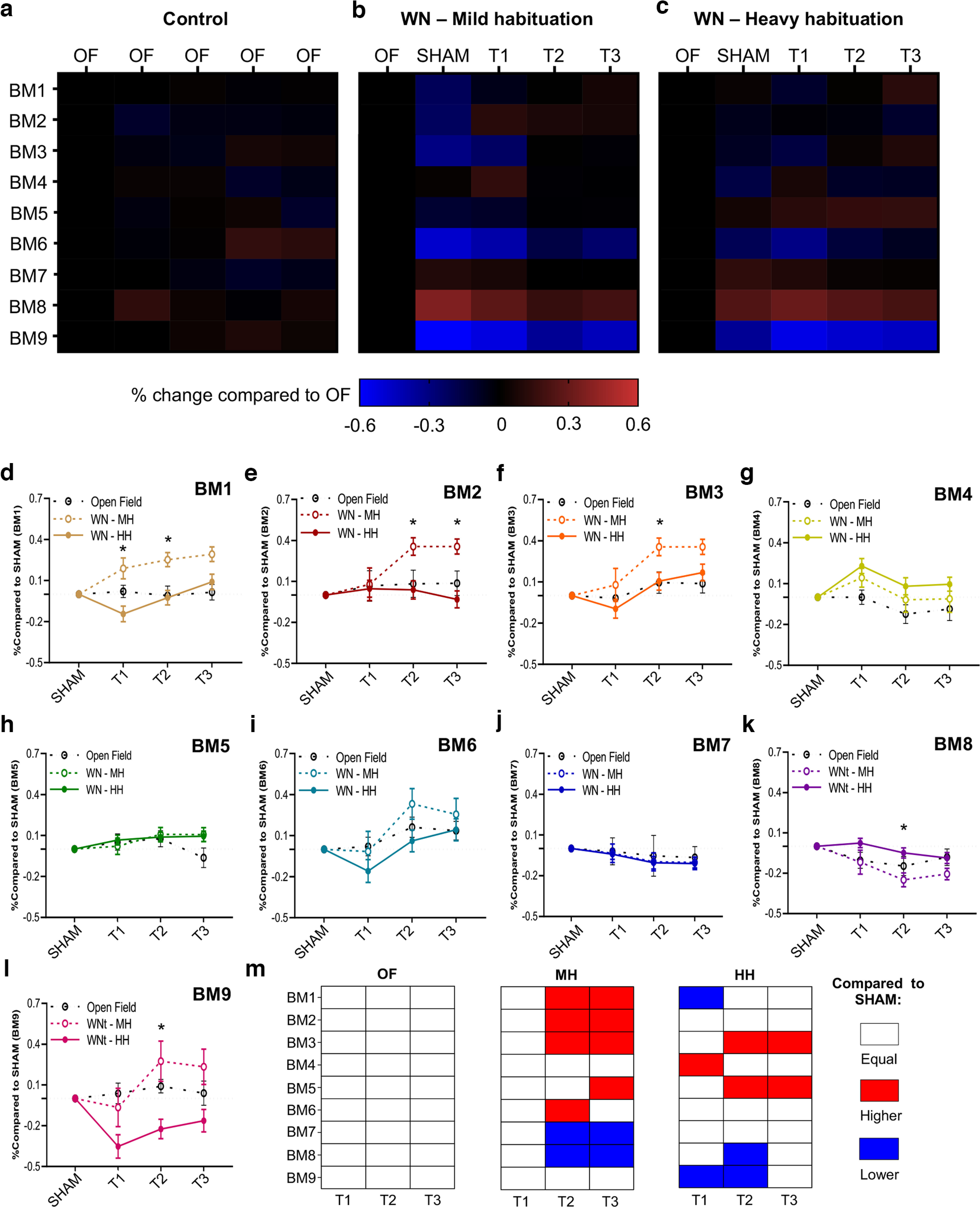
SEB3R discriminates stimulus-driven behavioral changes. ***a***, Control condition (open field, OF). ***b***, Whisker nuisance (WN) with heavy habituation (HH). ***c***, WN with mild habituation (MH). The percentage change in time spent in BMs varies depending on the test session and experimental conditions. The heatmap scale indicates the percentage change in the time spent in each BM normalized over the habituation session (baseline value = 0%); where the habituation session is the first of the five sessions in each of the three experimental conditions (control, WN-HH, and WN-MH). (Statistics are summarized in Extended Data Fig. 4-1.) ***d–l***, Each plot shows the percentage changes of BMs with respect to the SHAM session, for all three experimental conditions. Asterisks (*) indicate *p* < 0.05 between HH and MH. ***m***, Diagram recapitulating significant differences between the SHAM session and each stimulation trial. White = no difference. Red = significantly higher than SHAM. Blue = significantly lower than SHAM. (Statistics are summarized in Extended Data Figs. 4-2, 4-3, 4-4, 4-5, 4-6, 4-7, 4-8, 4-9, 4-10 for repeated measure and Extended Data Figs. 4-11, 4-12, 4-13 for between groups multiple comparisons). Error bars refer to SEM.

10.1523/ENEURO.0514-22.2023.f1-4 Extended Data Figure 1-4 Example of the output file Clustered.CSV provided at the end of step 1 and required to run step 3, for a representative mouse. OF in the file name indicates that this file belongs to an open field session. Download Figure 1-4, TIF file.

**Figure 5.**
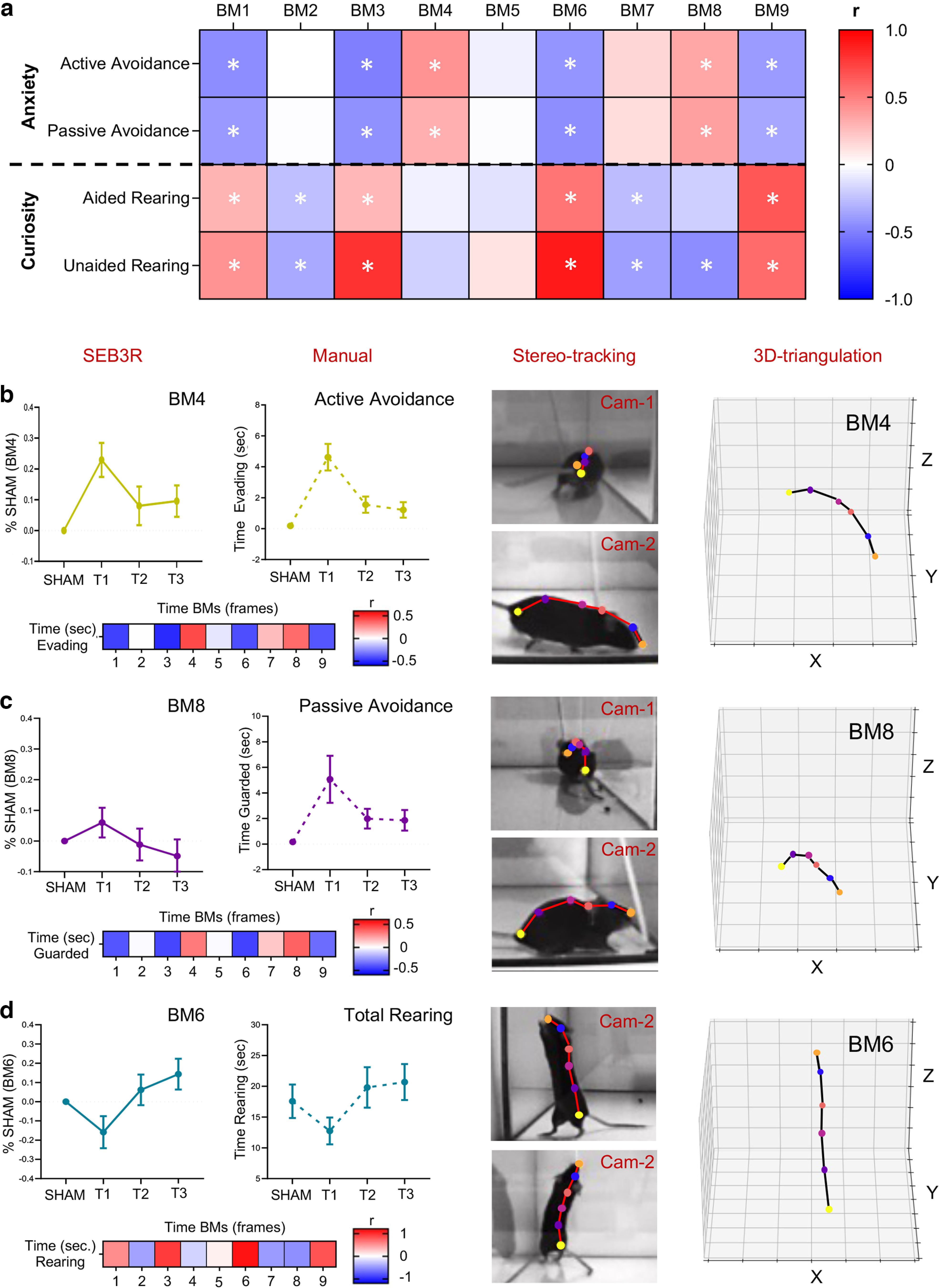
BMs correlate with discrete behavioral categories quantified by a trained user. ***a***, Heatmap showing correlation between BMs and behavioral categories. Color indicates strength and direction of Pearson’s *r* (summarized in Extended Data Fig. 5-1). Asterisk indicates significant correlation (summarized in Extended Data Fig. 5-2). ***b–d***, Left panels, Top charts, Correlated BMs and behavioral categories show a similar temporal progression. Left panels, Bottom charts, Heatmaps showing the strength and direction of the correlations between BMs and behavioral categories. Center panels, Representative frames corresponding to each BMs displayed in the left panel. Right panels, 3D triangulation of corresponding BMs. Error bars refer to SEM.

10.1523/ENEURO.0514-22.2023.f1-5 Extended Data Figure 1-5 Example of the final output of the pipeline provided for a single mouse (mouse no. 156). OF in the file name indicates that this file belongs to an open field session. Download Figure 1-5, TIF file.

**Figure 6.**
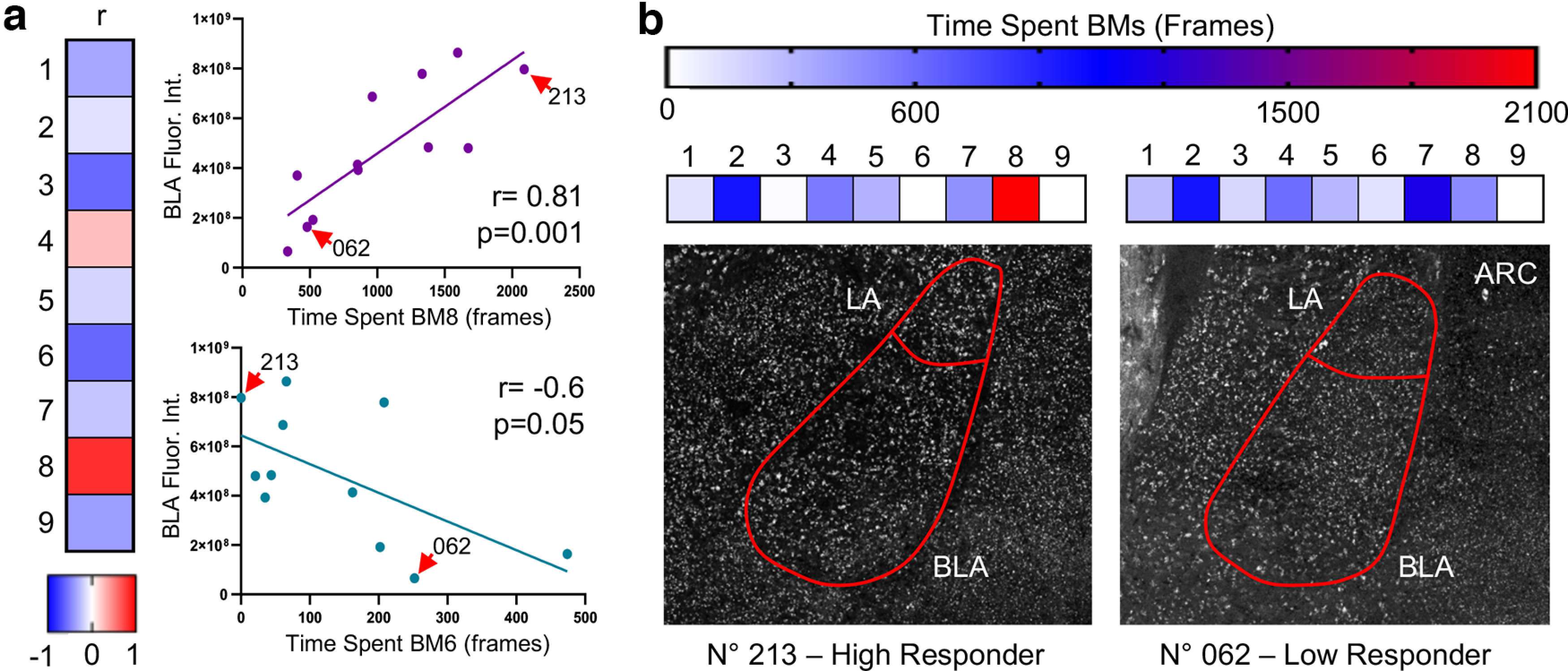
Stimulus-induced Arc protein expression in the amygdala. ***a***, Arc protein expression in BLA correlates positively with BM8 and negatively with BM6. Red arrows point out the subjects shown in ***b***). ***b***, Representative diagrams show one animal displaying elevated fearful response and high Arc staining (left, No. 213) compared with a second subject showing low fearful response and low Arc staining in BLA (right, No. 062). BLA, basolateral amygdala; LA, lateral amygdala. (Lack of correlation between BMs and LA is shown in Extended Data Fig. 6-1.)

10.1523/ENEURO.0514-22.2023.f1-6 Extended Data Figure 1-6 Example of the final output of the pipeline recapitulating the total number of frames assigned to each independent BM for a representative mouse (mouse no. 156) across the five consecutive sessions of the WN. Download Figure 1-6, TIF file.

## Materials and Methods

### Animals

All experimental procedures were performed in accordance with Italian and European directives (DL 26/2014, EU 63/2010) and were reviewed and approved by the University of Trento animal care committee and Italian Ministry of Health. Animals were housed in a 12/12 h light/dark cycle with unrestricted access to food and water. All killings for brain explant were performed under anesthesia and all efforts were made to minimize suffering. A total of 48 age-matched adult wild-type littermates of both sexes (weight 25–35 g) were used for the study. All mice were generated from our inbred CNTNAP2 colony with C57BL/6 background (Balasco et al., 2022b). Thirty-six mice were assigned to the whisker stimulation test, while 12 were assigned to the open field condition.

### 3D motion capture

A basic requirement for DLC tracking in 3D (DLC-3D) is to capture the subject motion using (at least) two video cameras in a stereo-configuration (Mathis et al., 2018; Nath et al., 2019). To this goal, two video cameras (ImageSource DMK27BUR0135 USB3-monochrome, equipped with TAMRON 13VM308ASIRII objectives) were secured, using a metal pedestal, on a board of laminated wood, in orthogonal positioning respect to each other (Fig. 1*d*). At the corner of the base, two aluminum guides were placed to ensure the stability of the cubic cage during the test. This allowed us to replace the experimental cage at every given animal, thus eliminating experimental biases because of the odor of other mice, that could potentially alter the emotional state of the test subject. Videos were acquired at a rate of 25 frames per second (fps) using the OBS-studio software, and edited using a commercially available software (OpenShot Video Editor), before being analyzed with DLC. For detail about how to use DLC-3D we recommend this link: https://github.com/DeepLabCut/DeepLabCut/blob/master/docs/Overviewof3D.md.

### Behavioral testing

For all experiments, mice were previously acclimated with the experimental setup for 4 d. For the first 3 d, mice were placed into the experimental cage (a fiberglass cubic box with open top; 25 × 25 × 20 cm) for 2 min. This time was extended to 10 min on day 4 (day before the test). This habituation scheme will be referred as heavy habituation (HH in figures and tables) for the rest of the manuscript. On the day of the test, mice were acclimated with the experimental environment for 5 min, before the beginning of the recordings. To assess the efficacy of our pipeline in discriminating between experimental conditions, a group of 12 mice underwent WN after only 2 d of habituation [referred to as mild habituation (MH) in figures and tables]. All experiments were performed in dim light.

### Open field

Mice were placed and left free to navigate the experimental cage for a total of 20 min. Video recordings followed the same chronological scheme of the WN to be able to compare the two conditions with each other (Fig. 1*e*, top).

### Whisker nuisance task (WN)

WN was performed with some variation compared with what previously described (Chelini et al., 2019; Balasco et al., 2022a). Before the beginning of the actual test, 3 min of the animal freely moving in the open field (OF) arena were videotaped as a baseline activity session. The testing phase was composed of four sessions, lasting 3 min each. In the first session (SHAM), a wood stick was introduced in the experimental cage, avoiding direct contact with the animal. The following three sessions (T1–T2–T3) consisted in stimulating mice’s whiskers by continuously deflecting vibrissae using the wooden stick (at a frequency of approximately three strokes per second; Fig. 1*e*, bottom).

To dissect the complexity of behavioral response, multiple behavioral categories were independently quantified by a trained observer. The identified categories, re-adapted from previous version of the test (McNamara et al., 2010; Fontes-Dutra et al., 2018; Chelini et al., 2019; Balasco et al., 2022a), included fearful and curious behaviors. Fearful behaviors were divided into active avoidance (time the animal spends actively moving away from the stick) and passive avoidance (time the animal spends in a defensive posture consisting in curved back, protracted neck, and stretched limbs or retracted in fully hunched posture; this measure also included the time in freezing). Curious behaviors were divided in aided rearing (when the animals, during a rearing action, leans on the arena’s walls investigating the surrounding environment) and unaided rearing (when the rearing is not supported by walls; this behavior usually occurs toward the center of the arena and is associated with stick exploration during WN).

### Tissue processing and immunofluorescence

Ninety minutes after the end of WN, mice were deeply anesthetized with isoflurane and killed by decapitation. Brains were excised, washed in 0.1% PBS and postfixed overnight in 4% paraformaldehyde (PFA), switched to a cryoprotectant solution (80% PBS, 20% glycerol with 0.1% sodium azide) and stored at 4°C. Cryoprotected brains were sectioned on a vibratome (Leica, VT1200) at 40-μm thickness. Serial sections were collected in 24 separate compartments and stored at 4°C in cryoprotectant solution.

Free-floating slices were rinsed three times in PBS (10 min each), then washed in PBS containing 0.2% detergent (Triton X-100, Fisher, AC215680010) for 30 min. Tissue sections were then incubated in blocking solution [2% bovine serum albumin (BSA), 1% fetal bovine serum (FBS) in PBS] for 3 h and then transferred to primary antibody solution [2% BSA, 1% FBS, 1:1000 dilution of primary antibody (rabbit anti-Arc/Arg 3.1; Proteintech, catalog 316 290-1-AP)] and incubated at room temperature for 24 h. Then, sections were rinsed three times in PBS (5 min each) and placed in a fluorophore-conjugated (Alexa Fluor Plus 488) secondary antibody solution [1:300 dilution of donkey anti-rabbit secondary antibody (Thermo Fisher Scientific, AB_2762833) in PBS] for 24 h. Sections were then washed 5 min in PB, mounted on superfrost slides, dried for 1 h and coverslipped with fluorescent mounting medium (Southern biotech 0100-01). Slides were stored at 4°C in the dark until use.

### Confocal microscopy and image analysis

A confocal laser scanning microscope Leica TCS-SP8, equipped with a HC PL APO 20× objective and interfaced with Leica LAS-X software was used to detect ARC immunolabeling in the lateral (LA) and basolateral (BLA) nuclei of the amygdala. Images were recorded at a resolution of 1024 pixels square, 400-Hz scan speed. Excitation/emission wavelengths were: 490/520 for Alexa-488 fluorophore. Acquisition parameters were set during the first acquisition and kept consistent for all the images. Corrected fluorescence intensity (CFI) was quantified using ImageJ software according to the formula: CFI = integrated density – (area size × mean fluorescence of background readings).

### Data collection and analysis

Data collection for all studies was conducted by investigators blind to experimental conditions. All statistical analyses were conducted using JMP-pro software (JMP Statistical Discovery LLC, 2023). For the purpose of this study, we have used three different experimental settings (open field, WN mild habituation and WN heavy habituation) to assess SEB3R ability to discriminate between experimental conditions. By consequence, the absolute time spent in BMs was not a reliably comparable measure, because of noticeable changes in the animal’s behavior during the SHAM session. These discrepancies are a direct consequence of the animal familiarity with the experimental setting (habituation regimen) or the lack of the stimulus in the control condition. To overcome this technical limitation, two separate normalization strategies where used. Time spent in BMs was normalized using the habituation session (first 3 min of videorecording) as a reference baseline using the formula: (number of frames during each test session – number of frames during habituation session)/(number of frames during each test session + number of frames during habituation session), to demonstrate SEB3R ability to discriminate between stimulus-independent and stimulus-driven changes. Similarly, to demonstrate SEB3R ability to discriminate between whisker-independent and whisker-dependent behaviors we normalized the time spent in BMs using the SHAM session as a reference with the formula: (number of frames during whisker stimulation session – number of frames during SHAM session)/(number of frames during whisker stimulation session + number of frames during SHAM session).

### SEB3R workflow

The SEB3R pipeline is summarized in Figure 1*f*. To run the MATLAB scripts, body-parts coordinates need to be first identified using DLC-3D. For instruction about DLC usage we suggest this link (https://github.com/DeepLabCut) and the relative literature (Mathis et al., 2018; Nath et al., 2019). As indicated on DLC-3D instructions, no major camera requirements are needed, as long as the videos can be reliably labeled to train the network. We recommend users to choose carefully the cameras acquisition rate depending on their experimental needs, as the final output of SEB3R will provide posture identification for each individual frame. Similarly, it is important to ensure both devices run at the same acquisition rate and are temporally synchronized. As for the neural network, for the validation experiment, we used ResNet-50, but we see no reasons the pipeline would not work with other options included in DLC. The body parts required by the analysis are (shown in Fig. 1*a–c*): nose, eyes, neck, mid back, lower back, and tail attachment.

After training your 3D network, analyze your videos and save the output tracking file in .CSV format. For each subject, place all the tracking files into a folder labeled with the animal identification (ID) number. Files should also contain the same ID in the name. Then place all subjects’ folders into the same mother-folder. It is recommended, for experiments composed of multiple sessions, to analyze videos from each session separately. Doing so, the pipeline will provide a separate output for each of the session, facilitating data analysis. In this case it is important for all DLC-3D files to contain the same number of frames analyzed.

#### SEB3R step 1

Identify meaningful postures in a single subject. Launch the script PoseExtraction by selecting your mother-folder. Assuming you have multiple tracking files for each subject (like in the case of an experiment with multiple sessions on the same mouse), the first step of SEB3R imports and combines the columns containing the *z*-coordinates of each subject. This step will ensure that postures identification is uniformed across various experimental conditions, limiting the risk of obtaining false-positive results. Then, from the *z*-coordinates, and within each frame, the linear distance between all the identified hotspot is calculated subtracting the values of each individual coordinate with the value of all the others. This will result in a 15-by-*N* matrix, with *N* = sum of the number of frames contained in the original coordinate files. This simple step eliminates most of the interindividual variability because of discrepancies in the animal dimensions, transforming the row coordinates into relative distances scaled by the animal body size. Using the relative distances matrix, the algorithm runs a *k-means* clustering using a customized version of the MATLAB function *kmean*: *kmean_opt* (De Landtsheer, 2019). This function uses the Elbow method, a statistical strategy to determine the number of *k-mean* clusters to choose (Thorndike, 1953). In our context, the advantage of this methods is to identify a “*K*” number of clusters preventing any input from the human user, thus achieving complete unsupervised identification. Each subject is processed independently, but the algorithm will sequentially run on all the files included in the mother folder, providing the results of *k-mean* clustering for each individual animal. The script runs for 5–10 min per subject, depending on your device, and outputs two folders containing a summary of the results of the clustering. Clusters identified in this step correspond to statistically relevant postures and will be recapitulated in an output file (MouseIDDistMean.CSV) containing (1) the animal ID in the first column; (2) the averaged distances between hotspots sorted by individual postures (columns 2–16); (3) the posture identification number for each row, on column 17 (Extended Data Fig. 1-1). A copy of all the MouseIDDistMean.CSV files for each subject will be saved in the folder named Mean Distances, within the mother folder. These are the files that will be used in step 2.

#### SEB3R step 2

Match postures identified within each subject to determine behavioral modules (BMs) replicated across the entire experimental group. Launch the script ModulesExtraction and select all the folder Mean Distances (containing the output files named MouseIDDistMean.CSV) within the mother folder. This script imports and combines all the averaged distance matrices for each subject and runs a second *k-mean* clustering, automatically matching postures identified in different animals. In this case, the user needs to choose the *k-number* of clusters to select to maximize BM representation of in the cohort. A window will pop-up asking the user to specify the *k-number* desired, that will correspond to the final number of BM (Extended Data Fig. 1-2). To this goal, it is recommended to use a *k = MIN(poses) + 1*, where *MIN(poses)* is the minimum number of postures identified in a single subject. As an example, in our validation experiment the lowest number of postures identified in a single subject was 8, hence we chose a *k = 9*. This crucial step converges highly similar poses (within and between subjects) into uniform BMs replicated across all (or most) animals, which can be used for comparisons, under the assumption that they are equally informative of the same behavioral state. The final number of BMs obtained with this method has minimal interindividual variability (Fig. 1*g*). Furthermore, we found no differences in the number of BMs identified in males and female subject (Fig. 1*h*,*i*), suggesting that the animal body size does not influence the detection of BMs. For all subjects, the matched pairs (posture-BMs) are recapitulated in the output file named ModulesAssigned.CSV (Extended Data Fig. 1-3) that will be directly saved in the mother folder. This file will be required to proceed with the third and last step.

#### SEB3R step 3

Assign BMs to each frame. Launch the script AssignModulesAndQuantify and select the mother folder and the ModulesAssigned.CSV file. The algorithm will import, one-by-one, the Clustered.CSV files generated in step 1. These files originate from the initial DLC-3D tracking and contain: the animal ID in column 1, the *z*-coordinates for each frame in columns 2–7, and the posture identifier (i.e., clusters identified in step 1) assigned to each frame in column 8 (Extended Data Fig. 1-4). Using the output of step 2 (ModulesAssigned.CSV), the postures contained in column 8 of Clustered.CSV are matched with the corresponding BMs, generating an output file identical to the Clustered.CSV, with the addition of a column 9 containing BMs identifying number for each frame (MouseID.CSV; Extended Data Fig. 1-5). The output files of this step will be saved in the folder Modules located within each subject subfolder (one file will be generated for each original DLC-3D tracking files input at the beginning of step 1). Thanks to this step, each frame is labeled with the appropriate BM, providing behavioral recognition on a timescale that depends on the original cameras acquisition frame-rate, allowing absolute flexibility in analyzing time-dependent changes in the behavioral flow. Figure 2 illustrates the nine BMs detected in our validation experiment. Finally, for each subject, the pipeline prints a file recapitulating the total number of frames assigned to each independent BM (FrequenciesMouseX.CSV; Extended Data Fig. 1-6), which can be used as an outcome measure to quantify the expression of different behaviors over consecutive sessions of the same test. In these files, each row corresponds to a BM and each column contains the number of frames assigned to that BM for each separate session of the test, according to the number of DLC-3D tracking files originally provided.

### Code accessibility

This code can run on any personal laptop on MATLAB-2020 and subsequent versions. The MATLAB code for SEB3R with the relative instructions are available on public repository GitHub, at the following address: https://github.com/gchelini87/SEB3R.

We also share the data resulting from our validation experiments on g-node platform as an aid to familiarize with the use of SEB3R: https://gin.g-node.org/gchelini/SEB3R_Validation_Data/settings.

## Results

Previous studies show that during the consecutive sessions of the WN the mouse attitude toward the stimulus (wooden stick) shifts drastically from a complex aversive response to relaxed and explorative (Balasco et al., 2022a). For this reason, this test was chosen to assess whether the BMs detected with SEB3R (Fig. 2*a–i*) could be used to reliably identify changes in stimulus-driven fearful and curious behaviors over an extended experimental session.

### SEB3R provides subsecond behavioral detection

Figure 3 shows that BMs can be reliably identified on a frame-by-frame resolution during the WN. All nine BMs are detected throughout the four sessions (sham, T1–T3) of the whisker stimulation task.

10.1523/ENEURO.0514-22.2023.f3-1 Extended Data Figure 3-1 Violin plots depicting the variability in the duration of behavioral bouts sorted by BMs. Note how exclusively curiosity-driven BMs (3-6-9) do not change between experimental conditions. The color code for each BM is the same used in Figures 2–5 and Extended Data Figure 4-1. Download Figure 3-1, TIF file.

We found that BMs duration is highly variable, and the majority of BMs events are composed of sequences of one to ∼20 frames, with a corresponding median of three frames (120 ms) that is shared by all three experimental conditions (Fig. 3*c*). Exceptionally we observed some longer bouts spanning from 400–806 frames in all three experimental conditions (see an example in Movie 1). Given our resolution of 25 fps (that is, each frame captures 40 ms of action), we wondered whether there was a lower threshold in the duration of BMs to be considered ethologically unreliable. To answer this question, we quantified the cumulative percentage of time spent in BM bouts shorter than three frames using the following formula: [(number of frames in 1-frame long bouts + number of frames in 2-frames long bouts) × 100]/(total number of frames) (Fig. 3*d*). To note, and in keeping with what shown in Figure 3*c*, manual quantification revealed that it is common to record behavioral bouts resolving within 100 and 200 ms, leading us to hypothesize that three frames might be the lowest reasonable threshold for reliable detection of ethologically relevant behaviors.

Movie 1. Example of the longest behavioral bout identified by SEB3R in the validation study; total length of the bout was 806 frames corresponding to ∼32 s. 10.1523/ENEURO.0514-22.2023.video.1

To our surprise, we found that sequences lower than 100 ms account for ∼11% of the total time in the WN (irrespectively to the habituation condition), while only ∼8% in the OF (Fig. 3*d*). More broadly, we found that BMs sequences are longer in the OF compared with both WN conditions (Fig. 3*c*; Kruskal–Wallis’s test: mild vs heavy habituation *p* = 0.49; mild habituation vs OF *p* < 0.0001; heavy habituation vs OF *p* < 0.0001). Moreover, this effect is abolished in curiosity-driven BMs 3-6-9 (Extended Data Fig. 3-1). While our data do not allow to conclude that bouts lower than 100-ms capture complete behavioral sequences, the discrepancy observed between OF and WN suggests that the velocity of behavioral action increases in response to the invasive stimulation. This indicates that even the lowest detectable BMs sequences retain substantial biological valence. Speculatively, we interpret these short bouts as transitioning frames from one BM to another, rather than full behavioral sequences. However, since we do not have instruments to definitively establish this claim, we have included them as part of the total time of BMs expression. We believe this kind of analysis could be reliably used as an additional metric to investigate qualitative behavioral differences; hence, we have included a script to SEB3R toolbox to automatically extrapolate BMs sequence duration (SequenceDuration.m).

### BMs identified using SEB3R discriminate stimulus-driven changes in mouse behavior

To assess SEB3R ability to discriminate between experimental conditions, we compared the percentage change in time spent in each BM during the habituation phase with the four consecutive sessions of the test. Predictably, no significant differences were observed at any timepoint in the open field (OF) condition (Fig. 4*a*). Conversely, we identified significant changes in the heavy habituation (BM1, prob > χ^2^ > 0.02. BM3, prob > χ^2^ > 0.005; BM4, prob > χ^2^ > 0.0108; BM5, prob > χ^2^ > 0.011; BM6, prob > χ^2^ > 0.023; BM8, prob > χ^2^ > 0.0001; BM9, prob > χ^2^ > 0.0001; Fig. 4*b*) and mild habituation (BM1, prob > χ^2^ > 0.0013; BM3, prob > χ^2^ > 0.0022; BM6, prob > χ^2^ > 0.0112; BM8, prob > χ^2^ > 0.0001; BM9, prob > χ^2^>0.0001; Fig. 4*c*) groups at several different timepoints (descriptive statistics and *p*-values of the Friedman’s test for nonparametric repeated measure relative to this analysis are summarized in Extended Data Fig. 4-1). Notably, the majority of the BMs (seven out of nine) showed changes starting from the SHAM session, when the stimulus (stick) was introduced in the arena, but before the beginning of the whisker stimulation. This data demonstrates that the exposure to a novel stimulus, although not tactile, is sufficient to trigger a meaningful change in mice behavioral state. Then, to specifically assess the whisker-dependent changes in behavior, we normalized BMs intensity as percentage variation from the SHAM and compared the three consecutive sessions of whisker stimulation (T1–T2–T3) with the baseline value (SHAM = 0, after normalization), for all three experimental conditions. Data resulting from the control condition (open field, OF) were normalized in analogous way, using the second session of acquisition in replacement for the SHAM. As a reference, we established that there were no changes in the open field, in none of the timepoints compared with the baseline (T1–T2–T3; Fig. 4*d–l*). In the mild habituation group, instead, significant increase in the expression of BM1 (T2 and T3, *p* < 0.0001), BM2 (T2, *p* = 0.0006. T3. *p* < 0.0001), BM3 (T2, *p* < 0.0001. T3, *p* = 0.0002), BM5 (T3, *p* < 0.0034), BM6 (T2, *p* = 0.0077), and BM9 (T2, *p* < 0.0077) were found, while a significant decrease was observed for BM7 (T2 and T3, *p* < 0.0034) and BM8 (T2, *p* = 0.0006, T3, *p* < 0.0001). Finally, in heavy habituation, a significant increase was found in BM3 (T2, *p* = 0.0084. T3, *p* = 0.0016), BM4 (T1, *p* = 0.0016) and BM5 (T2 and T3, *p* = 0.006), while a significant decrease was found in BM1 (T1, *p* = 0.0084), BM8 (T2, *p* = 0.0036) and BM9 (T1, *p* < 0.0001. T2, *p* = 0.0005). Descriptive statistics and the results of Friedman test with Wilcoxon test for *post hoc* repeated measure multiple comparisons are summarized in Extended Data Figures 4-2, 4-3, 4-4, 4-5, 4-6, 4-7, 4-8, 4-9, 4-10; significant differences with SHAM are graphically recapitulated in Figure 4*m*. Moreover, we found that the mild habituation condition showed significant increase in the expression of BM1 (T1, *p* = 0.01. T2, *p* = 0.0027), BM2 (T2, *p* = 0.008. T3, *p* = 0.0004), BM3 (T2, *p* = 0.008), BM8 (T2, *p* = 0.008), and BM9 (T2, *p* = 0.005) compared with heavy habituation (asterisks in Fig. 4*d*,*f*,*k*,*l*; descriptive statistics and results of Mann–Whitney test with Steel–Dwass nonparametric multiple comparisons are summarized in Extended Data Figs. 4-11, 4-12, 4-13). Extended Data Figure 4-14 shows the absolute time spent in BMs before normalization.

10.1523/ENEURO.0514-22.2023.f4-1 Extended Data Figure 4-1 Descriptive statistics and *p*-values of the Friedman’s test for all experimental conditions. Download Figure 4-1, XLSX file.

10.1523/ENEURO.0514-22.2023.f4-2 Extended Data Figure 4-2 Results of Friedman’s test with Wilcoxon test *post hoc* multiple comparisons for nonparametric repeated measures for BM1, across all three experimental conditions. Significance level (α = 0.05) set at *p* < 0.0084 after Bonferroni correction. Download Figure 4-2, XLSX file.

10.1523/ENEURO.0514-22.2023.f4-3 Extended Data Figure 4-3 Results of Friedman’s test with Wilcoxon test *post hoc* multiple comparisons for nonparametric repeated measures for BM2, across all three experimental conditions. Significance level (α = 0.05) set at *p* < 0.0084 after Bonferroni correction. Download Figure 4-3, XLSX file.

10.1523/ENEURO.0514-22.2023.f4-4 Extended Data Figure 4-4 Results of Friedman’s test with Wilcoxon test *post hoc* multiple comparisons for nonparametric repeated measures for BM3, across all three experimental conditions. Significance level (α = 0.05) set at *p* < 0.0084 after Bonferroni correction. Download Figure 4-4, XLSX file.

10.1523/ENEURO.0514-22.2023.f4-5 Extended Data Figure 4-5 Results of Friedman’s test with Wilcoxon test *post hoc* multiple comparisons for nonparametric repeated measures for BM4, across all three experimental conditions. Significance level (α = 0.05) set at *p* < 0.0084 after Bonferroni correction. Download Figure 4-5, XLSX file.

10.1523/ENEURO.0514-22.2023.f4-6 Extended Data Figure 4-6 Results of Friedman’s test with Wilcoxon test *post hoc* multiple comparisons for nonparametric repeated measures for BM5, across all three experimental conditions. Significance level (α = 0.05) set at *p* < 0.0084 after Bonferroni correction. Download Figure 4-6, XLSX file.

10.1523/ENEURO.0514-22.2023.f4-7 Extended Data Figure 4-7 Results of Friedman’s test with Wilcoxon test *post hoc* multiple comparisons for nonparametric repeated measures for BM6, across all three experimental conditions. Significance level (α = 0.05) set at *p* < 0.0084 after Bonferroni correction. Download Figure 4-7, XLSX file.

10.1523/ENEURO.0514-22.2023.f4-8 Extended Data Figure 4-8 Results of Friedman’s test with Wilcoxon test *post hoc* multiple comparisons for nonparametric repeated measures for BM7, across all three experimental conditions. Significance level (α = 0.05) set at *p* < 0.0084 after Bonferroni correction. Download Figure 4-8, XLSX file.

10.1523/ENEURO.0514-22.2023.f4-9 Extended Data Figure 4-9 Results of Friedman’s test with Wilcoxon test *post hoc* multiple comparisons for nonparametric repeated measures for BM8, across all three experimental conditions. Significance level (α = 0.05) set at *p* < 0.0084 after Bonferroni correction. Download Figure 4-9, XLSX file.

10.1523/ENEURO.0514-22.2023.f4-10 Extended Data Figure 4-10 Results of Friedman’s test with Wilcoxon test *post hoc* multiple comparisons for nonparametric repeated measures for BM9, across all three experimental conditions. Significance level (α = 0.05) set at *p* < 0.0084 after Bonferroni correction. Download Figure 4-10, XLSX file.

10.1523/ENEURO.0514-22.2023.f4-11 Extended Data Figure 4-11 Results of Steel–Dwass multiple comparisons for trial 1 of the WN, between the three experimental conditions (session 3 of the open field chronologically corresponds to T1 of WN). Download Figure 4-11, XLSX file.

10.1523/ENEURO.0514-22.2023.f4-12 Extended Data Figure 4-12 Results of Steel–Dwass multiple comparisons for trial 2 of the WN, between the three experimental conditions (session 4 of the open field chronologically corresponds to T2 of WN). Download Figure 4-12, XLSX file.

10.1523/ENEURO.0514-22.2023.f4-13 Extended Data Figure 4-13 Results of Steel–Dwass multiple comparisons for trial 3 of the WN, between the three experimental conditions (session 5 of the open field chronologically corresponds to T3 of WN). Download Figure 4-13, XLSX file.

10.1523/ENEURO.0514-22.2023.f4-14 Extended Data Figure 4-14 ***a–i***, Time spent in BMs for the three experimental conditions. Note how, because of the differences in experimental settings, the absolute time spent in BMs during the SHAM session changes across experimental groups, rendering the interpretation of stimulus-driven behavior inconsistent. This discrepancy can be overcome by using the SHAM session as a baseline reference to normalize and express the relative time spent in BMs as percentage change compared to SHAM in response to the whisker stimulation. Download Figure 4-14, TIFF file.

Altogether, these findings confirm that SEB3R classification successfully identifies time-driven and stimulus-driven changes in the expression of specific behaviors, discriminating between different experimental conditions.

### BMs are informative of the mouse emotional response to whisker stimulation

To evaluate SEB3R reliability in identifying dynamic changes in the mouse emotional state, we assessed the linear correlation between the time spent in each BM and the time spent into discrete behavioral categories quantified by a manual user during the WN. We observed that behaviors indicative of fear and anxiety had a strong positive correlation with BMs 4 and 8, while being negatively correlated with BMs 1-3-6-9 (Fig. 5*a*). More specifically, among fear-related BMs, BM4 showed stronger correlation with evasive behaviors (*r* = 0.42, *p* = 0.0002; Fig. 5*b*; Movie 2), while BM8 showed stronger association with passive avoidance response (*r* = 0.36, *p* = 0.001; Fig. 5*c*; Movie 3). A mirrored trend was observed for explorative rearing, where strong positive correlations were found for BMs 1-3-6-9, while negative correlations was observed for BMs 2-7-8 (Fig. 5*a*). As an example, Figure 5*d* shows correlation between BM6 and total rearing time (*r* = 0.92, *p* < 0.0001; Movie 4). No correlation was found between BM5 and the categories assessed (Fig. 5*a*), likely indicating that this specific BM relates to an emotionally neutral behavior such as spontaneous spatial navigation (Pearson’s correlation coefficients are summarized in Extended Data Fig. 5-1; *p*-values of the correlations are summarized in Extended Data Fig. 5-2). These data suggest that BMs identified by SEB3R can be reliably used as a proxy to isolate multiple emotionally charged behaviors.

10.1523/ENEURO.0514-22.2023.f5-1 Extended Data Figure 5-1 Coefficients of Pearson’s correlations between BMs and behavioral categories. Download Figure 5-1, XLSX file.

10.1523/ENEURO.0514-22.2023.f5-2 Extended Data Figure 5-2 *p*-values of the Pearson’s correlation between BMs and behavioral categories. Download Figure 5-2, XLSX file.

Movie 2. Example of a representative cluster of frames identified as BM4 taken from one of the original video files. BM4 was shown to be mostly correlated with active avoidance response. 10.1523/ENEURO.0514-22.2023.video.2

Movie 3. Example of a representative cluster of frames identified as BM8 taken from one of the original video files. BM8 was shown to be mostly correlated with passive avoidance response. 10.1523/ENEURO.0514-22.2023.video.3

Movie 4. Example of a representative cluster of frames identified as BM6 taken from one of the original video files. BM6 was shown to be mostly correlated with unaided rearing. 10.1523/ENEURO.0514-22.2023.video.4

### Fear-associated BM8 finds direct neural correspondence in the basolateral amygdala

To further provide proof-of-principle evidence of the biological relevance of our behavioral classification, we asked whether BMs could be mapped within specific brain areas. We therefore assessed the association between the activation of the amygdaloid complex with freezing-associated BM8 (as shown in Fig. 5*a*,*c*) in the mild habituation group, aiming to find correspondence with anxiety-related behaviors. To probe for neural activation, we used immunofluorescent labeling of ARC, a protein coded by an immediate early gene whose expression in the amygdala is activated by stressful stimuli (Dirven et al., 2022). We quantified the fluorescent intensity within the lateral (LA) and basolateral (BLA) nuclei. Strikingly, in BLA, we found that ARC expression was positively correlated with BM8 selectively (*r* = 0.81, *p* = 0.001; Fig. 6*a*,*b*). To the contrary, BLA ARC expression was negatively correlated with BM6 (*r* = −0.61, *p* = 0.05; Fig. 6*a*), which was identified as indicative of explorative behavior. No significant correlation was found between LA ARC expression and all BMs (Extended Data Fig. 6-1). These findings suggest that blindly identified BMs correspond to biologically relevant instances represented within specific neural circuits.

10.1523/ENEURO.0514-22.2023.f6-1 Extended Data Figure 6-1 Arc protein expression in lateral amygdala does not show correlation with BMs. Download Figure 6-1, TIF file.

## Discussion

In this work, we used a new-generation software for mouse body tracking combined with a strategy for automated posture detection to achieve successful and biologically relevant deconstruction of the mouse body language during freely moving behavior. This approach eliminates biases intrinsic of subjective rating strategies while significantly reducing the time required for quantification. Moreover, thanks to the frame-by-frame temporal resolution (Fig. 3), it offers the flexibility to study behavioral changes in selected time-windows as well as for extended periods. This feature renders our approach well-suited to ask questions beyond the simple categorical quantification looking at presence of absence of a behavioral outcome, allowing investigation of qualitative features such as complexity, persistency, and flexibility of the response to a stimulus. In addition, it detects the fluctuations of ethologically relevant behaviors in real time; as an example, SEB3R could be adapted to quantify freezing in fear-conditioning paradigms or to track pursuit during hunting. Finally, our approach would allow to investigate the neural correlates of specific behavioral hallmarks by combining behavioral parcellation with *in vivo* electrophysiological recordings or optical imaging. To this point, we showed that the frequency of BM8, putatively identified as associated with passive avoidance response (Fig. 6), was strongly correlated with activity-dependent ARC immunolabeling in the BLA, thus suggesting that BMs are not only valuable proxies for behavioral interpretation, but have a well-defined neuroanatomical location to be exploited for *in vivo* studies.

Our validation experiments focused on a whisker-guided response (WN), a task that triggers complex and ambivalent reaction in wild-type mice (McNamara et al., 2010; Fontes-Dutra et al., 2018; Chelini et al., 2019; Balasco et al., 2022a). One major limitation of previous versions of this approach consists in the fact that most manually quantified behaviors show virtually the same temporal progression depending on their biological valence; anxiety-related behaviors dramatically increase during the first trial and are followed by a sharp decline in the second and third trials because of adaptation; an opposite tendency is observed for explorative categories (Chelini et al., 2019; Balasco et al., 2022a). Thanks to SEB3R and its unbiased extraction of BMs, we described independent behavioral categories characterized by their own temporal fluctuation as well as specific intrinsic interindividual variability, demonstrating a more scrupulous selection of behavioral states. At the same time, the automated quantification retained the capacity of discriminating between groups characterized by different affective states, as testified by the differences observed between heavy habituation and mild habituation (Fig. 4). Given the parallelisms and discrepancies observed with the manual quantification, it is important to clarify the divergent nature of BMs with respect to traditional behavioral characterization. Especially for anxiety-related behaviors we have found significant correlation with behavioral categories, but the strength of the correlation was relatively weak (<0.5; Fig. 5*a–c*). This is not only because of the unavoidable noise included in both measurements, but also to the fact that the two measures are not directly overlapping. BMs are, by definition, indirect measures that can be used as a proxy to infer emotional states, they are not the behavioral state itself. By the same principle, correlation between manually and automatically quantified rearing is virtually perfect, as in that case the visual observer is directly quantifying the posture rather than a more complex behavioral construct. However, the remarkable similarities between automated and manual quantification, as in the case of the temporal dynamic of BM4 and its correspondence with evasive behaviors (Fig. 5*a*,*b*), confirm the efficacy of this computational approach in detecting emotion-driven behaviors, even in the context of a mildly emotionally loaded task, such as whisker stimulation. Thus, SEB3R promises to be an effective tool to study affective responses in tasks with high emotional salience such as fear conditioning.

This work aligns with recent advancement in system neuroscience and a variety of novel computational methods to study animal behavior (Wiltschko et al., 2015; Mathis et al., 2018; Nath et al., 2019; Dolensek et al., 2020; Bohnslav et al., 2021; Hsu and Yttri, 2021; Karashchuk et al., 2021; Luxem et al., 2022). Given this context, it is worth mentioning the advantages and limitations of this method. The main difference between SEB3R and other methods resides in its low computational complexity. *k-mean* cluster analysis is a relatively simple approach that does not compare in efficacy with emerging mathematical models used in other approaches. As a result, behavioral segmentation achieved with SEB3R is limited compared with previous studies (Wiltschko et al., 2015; Dolensek et al., 2020). By contrast, SEB3R’s reductionist approach degrades the behavioral complexity by forcingly compressing some datapoints in an array of interpretable behavioral modules, allowing the user to achieve a meaningful decoding of behavioral flow, to be used for translational inference. Another advantage of SEB3R stands in its technical feasibility. After obtaining behavioral tracking with DLC-3D, which has minimal technical needs per se (Mathis et al., 2018; Nath et al., 2019), the pipeline runs on any personal or professional device, without the need of a strong background in programming or computational skills. Thanks to minimal technical requirements of SEB3R we aim to provide the scientific community with a tool that uniforms behavioral segmentation across research laboratories, allowing to investigate the affective content of behavior in freely moving mice in multiple experimental settings. Nonetheless, SEB3R’s simplistic approach leaves room for future improvements. For instance, by discretizing the current classification it would be theoretically possible to include additional principal components to the cluster analysis, improving precision and specificity of the behavioral detection. In conclusion, the method proposed here will improve the translational validity of several behavioral tests by reducing quantification biases, ensuring replicability (within and beyond different laboratories), and expanding the number of behavioral categories to be analyzed. Finally, by applying novel system neuroscience approaches to a traditional ethology-based behavioral observation, we prove how the convergence of these two disciplines can contribute the study of the brain and its disorders.

## Synthesis

Reviewing Editor: Mark Laubach, American University Decisions are customarily a result of the Reviewing Editor and the peer reviewers coming together and discussing their recommendations until a consensus is reached. When revisions are invited, a fact-based synthesis statement explaining their decision and outlining what is needed to prepare a revision will be listed below. The following reviewer(s) agreed to reveal their identity: Eric Yttri.

Please see the reviews below and consider revising your manuscript to address all points that were raised.

Many of the files that were uploaded as supplementary files are either screenshots of tables or Excel spreadsheets with data. They are not figures. Perhaps all of these files can be provided in a common format, *.xlsx, and uploaded in a zipped archive.

Reviewer 1

I thank the authors for the attention and effort to greatly improve the manuscript. There are some major issues remaining but I am very impressed with their progress.

--- Stats ---

This was a major hold up for me when reviewing the paper and things are much improved. Thank you! I had a few comments on some critical remaining issues.- It is still unclear why delta sham/sham should be used. Would the authors please make their reasoning clear? In any number of behavior papers I can think of, the raw values are shown.- This points to a related issue. Somewhere we NEED the raw values. I’d argue that 4a-c are great to summarize the degree of change, but the plots in 4d-i should have real values. This is also CRITICAL because we cannot determine if some of the effects may be due to vagaries in the shams themselves. It shouldn’t be the case and I don’t presume it is, but we just can’t tell.- (I thank the authors for putting in the formula, so this is more minor, but is it really delta sham? I’d have said effect/sham, as the sham isn’t changing, aka doesn’t have a delta.)

- As far as I can tell, 4a-c and 4d-i are summaries of the same data - normalized slightly differently I think? If not, please make it clear how they are different. If they are the same relative data however, they have very different trends. for instance, in 4c, MH-BM1 is quite low for sham (blue), then higher for T1(black), but slightly reduced from T1 in T2 (dark blue). In 4d, T2 is greater than T1. Can the authors make it clear how this could be? The legend/text are quite sparse in terms of details that can help me parse this. Again, normalizing to OF in a-c and to sham(?) in d-i does not make things easier.

- Line 282. It’s still somewhat unclear to me what ‘baseline’ is. “Δsham/sham against the baseline (Δsham/sham during SHAM session)”. Could the authors please clarify? this is some method to incorporate both the sham and the control data??

- Maybe more minor, but Friedman’s test is not often used. It may be beneficial to briefly state in Results that it is a nonparametric test used to compare multiple dependent groups. Along those lines, I do not understand line 297: ‘Friedman test with Wilcoxin multiple comparisons’. Please explain?

- In 4a, please replace % time with % change as previously requested. This balances out the range, e.g. a change from 100 to 50 or to 200 are equal in magnitude (otherwise, the positive change has infinitely more range compared to the 0-100% of a negative change). I’d also change the legend in the figure to % change in time spent in BM. This also means that the stats on 4d-i are still invalid.

- I thank the authors for being thorough in their new stats reporting. For readability, they may want to use text to more summarize statistics. Exact values, particularly SEMs, can be found in the figures and supplemental tables. Instead, I’d strongly encourage something like the following (beginning on line 286:” ... significant increase in the expression of BM1 (p<0.0001, T2 and T3), BM3 (p<0.0005, T2 and T3), BM5 (p<0.005, T3), BM6 (p<0.01, T2)... while a significant decrease was observed for BM7 (p<0.05, T2; p<0.005 T3)”

- I cannot think of a way how, in Line 290, a significant difference can occur if the the SEM is twice the size of the effect: “BM7 (T2, mean=-0.07, SEM=0.15, p<0.034”. Please help my understanding??

- Figure 3 - The video sampling rate is 25hz. Some of these behaviors seem to only be one hash mark in duration. 1) please indicate in the figure legend if 1 hash = 1 frame2) in text, please indicate if behaviors were allowed to be 40ms (1/25 of a second) long, and if so, why this is reasonable. I apologize if I missed some details where this was explained.3) Please include a violin plot of durations for each BM and state the median durations in the text - duration being how many consecutive frames (e.g. if the frame labels were A A B A B A C C C, the mean durations would be A=53.3ms, B=40ms, C=120ms; assuming 40ms per frame) Fig 3 would be a good place for this. This is especially important given that in the example data, behaviors 3,4, 6 and 9 seem to have mean durations well under 100ms. Again, I apologize if I missed something.

- Line 268-272, I am not familiar with this

- Readability will be GREATLY increased if MH and HH are replaced by mild and heavy habituation in the main text.

I have no idea what this means (line 54-55) “To do so, we leveraged the innate human ability of visually discriminating some instances of the mouse emotional state.”Clear and simple language is your friend here. Same for ‘novel and renowned tools’ in the abstract and elsewhere.

- Line 272-273 - I do not understand this text.

- Minor:I appreciate 1e being included to explain exp design, but it confuses me still. I’d recommend removing the cameras - for space and because the one camera looks like it is from below. I’d also just have one title of ‘open field’ on the top and no mention of open field on the bottom, just sham and wnt.

- Try matching the pinks and blues in 1h and 1i?

- Wiltschko 2015 does not fit the description (it doesn’t use pose data / DLC).

- Figures are greatly improved. still some mistakes on figures, e.g. 4h has no readable y axis label, and the delta sham is hard to read (for this, just add a space)

- 4b,c - elsewhere you list Open field, MH, then HH. Why flip the order here? Please swap location of b and c.

- fig 4 legend, line 514: Please remove “The time spent in each BM changes drastically depending on the test session and experimental conditions.” This is a qualitative statement that would be summarized much better with the actual stats provided. More importantly, I strongly disagree that EACH BM changes drastically. HH BM2 shows little change.

- There are several places where additional proofreading will help.

- double check line 289. Though possible, two tests with exactly p=0.0077 would be a rare occurance. Similarly, in the following line, why have 0.034 and 0.0034?

- Most extended data figures are actually tables. I’ll leave it up to the authors and eNeuro to figure out the appropriate way to share this information. One possible idea would be to color code (thick black text for the values that are sig, lighter and lighter for non sig) to help parse all of these data. Even if just 2 colors for sig / non-sig, or 4 for very sig /sig / trending / non-sig. This is entirely optional though.Along these lines, figuring out which files went with what was very difficult. Files were named after videos, though this might have been done on eNeuro’s behalf.

Reviewer 2

The updated manuscript is reframed to provide a more wide-ranging applicability for the use of their tool, addressing the gap of what to do with / how to analyze data from deeplabcut. The authors provide a method that replaces manual behavior scoring in attempts to quantify behavior.

The manuscript reads much more clearly now, with more straightforward instructions for how the tool can be used, and a greater wide-reaching audience for different types of behavioral tasks/tests.

I think there should still be some re-framing in explicitly detailing how this tool is filling in the knowledge gap of what to do with data from deeplabcut and how to quantify the output, and also in comparison with other tools out there that have a very similar purpose.

I am still concerned about the similarity of comparison between manual scoring and the tool; for example the error bars are somehow greater with the tool as opposed to manual scoring, but possibly this can just be addressed in the results text as these two measures are hard to directly compare.

## Author Response

First, your research pipeline is specific to the whisker nuisance test. Can it be used for other kinds of studies? If so, please expand on this in the paper and include examples of using the pipeline for at least one other kind of neuroscience experiment.

1) We replaced the name from “Whisker nuisance test in 3D for rodents (WNt3R)” to “stimulus-evoked behavioral tracking in 3D for rodents (SEB3R)”, to clarify that our pipeline can be extended to other experimental paradigms.

2) The abstract, introduction and discussion have been substantially revised to highlight the broader applicative purposes of SEB3R. Doing so, we included the experimental work done with the WNt as a mean to and end validate the pipeline efficacy.

3) We emphasized the temporal resolution of SEB3R (in the methodological description and in the discussion). A new figure was included (Fig.3) to strengthen this point. We believe adding this detail will help other investigators employ SEB3R for other applications such as during fear conditioning experiments (as mentioned in the discussion).

4) We changed the title of the manuscript.

5) To make it more broadly available to the scientific community, we substantially restructured SEB3R code to improve flexibility and automation. The new code is already available on GitHub. Second, the reviewers perceived tension between reporting your analysis pipeline and reporting on experiments done using it. The feeling was to focus revisions on reporting the tool, especially in light of some concerns raised about your use of statistics (potential incorrect use of t-test, lack of correlational measures, many multiple comparisons without evidence of corrections performed).

1) We have included extensive methodological description in the methods section of the manuscript (as specified below). The data obtained with the WNt were repurposed to validate the pipeline efficacy (as specified above).

2) Following Reviewer 1 suggestions, we performed more appropriate statistical testing that is now reported both in the main text (where appropriate) and recapitulated in tables included in the extended data. Third, consensus was that the first point of Reviewer 2 is most important to address: “Several details should be included to provide readers with step-by-step walk-through of the pipeline. First, a schematic or diagram of the automation pipeline should be included, to provide readers with an idea of the analysis. This is attempted in Figure 1F, but is quite vague. An updated version of this schematic should describe that these are all being performed by the pipeline itself and not manually by the experimenter. Relatedly, a screengrab or description of what Matlab is producing at each stage/level of the pipeline should be included. There’s some nice detail in the supplement on the instruction manual for the toolbox that can be included in the methods as well (such as camera resolution, etc).”

As suggested:

1) The methods section now includes a step-by-step guide to the pipeline.

2) We modified the scheme in Figure 1, adding some details, to help the readers follow the multiple steps of SEB3R.

3) We provided a graphical abstract to help readers navigate the paper.

4) We included step-by-step screenshots of the MATLAB outputs as extended figures 1-6.

Reviewer 1

The authors present a method for automated segmentation of behavior using the output of deeplabcut 3d. This method is specifically applied within the context of the whisker nuisance test, a useful metric for the study of ASD and anxiety. Plenty of work has been done here. However, I found the manuscript very hard to follow and to gain information from. Although it is posed as a methods paper, I could find only superficial discussion of the actual methods used and minimal quantification of performance - skipping to linking it with other metrics like human rater categories and BLA activation. I would encourage the authors to focus. Refine the document. Think about what is most important. Although the BLA measures are interesting and provide a potentially informative ‘ground truth’ for the algorithmically defined behavioral groups, I’d much rather have a better account of how the BMs came to be and really what they mean. All the information could be great here, but I’d prioritize expanding the first half of the paper in a way that is approachable to an outside reader. Finally, the overall applicability/value is unclear from their text. From the authors description, only one lab seems to be using the WNt approach. Even with considerable effort, as a manuscript, I would want to read something that is of interest beyond just one lab.

Major:

Having tables for all of the comparisons / stats is helpful, however, it would be much better to have the means, sem’s and/or p values in the text for anything that the authors want to make a point about. It is also not made clear if multiple comparison corrections have been applied, and if so, how and using what methods. it is inappropriate use a t-test on delta f over f. t tests assume a continuous parametric distribution. percent difference is neither contiuous (you can’t be negative) nor continuous 0.5 is a having of the original 1.0value, while 2.0 is a doubling.s4, I could not find it stated what statistical test was used to determine significance.

- Statistical analysis was performed according to the reviewer’s feedbacks.

Means, SEM and p-values are now reported in the main text when relevant. Full extension of the statistical analysis is reported in tables within the supplementary materials. (also addressed in response to main point n.2)

Providing an account of the state/context of the field in which a development exists is very useful. I found this aspect of your paper to be quite sparse (save for the many, repeated citations of “Balasco et al, 2022, Chelini et al, 2019, Fontes-Dutra et al, 2018”). There are several tools worth comparing to for computational structure, if not performance, including VAME, B-SOID, DeepEthogram, or FaceMAP.

- We have included mention, with relative references, to other similar tools in the introduction and discussion. We discuss advantages and limitations in the discussion. Along these lines, the Wnter3r workflow is remarkably similar to other approaches. A process flow is provided, but other details are scant, the first paragraph of results. I recognize that this would be available to some extent on the github page, but as a methods-first manuscript, much more needs to be provided to the reader. I recommend checking out the methods/publications above for the depth of their explanation.

- The methods section now includes a step-by-step guide to the pipeline. Additionally, we added some details to the image (in Figure 1) that shows the workflow of the pipeline and provided a graphical abstract to help readers navigate the paper. As suggested, we included step-by-step screengrabs of the MATLAB outputs as extended figures 1-6. We discuss advantages and limitations in the discussion. (also addressed in response to main point n.3) Figure text is quite pixelated and is of different sizes (some far too small), making it difficult to read. More of an opinion, I would recommend against the boxing of panels within a figure.

- We have fixed the images according to eNEuro standards (300 dpi) and removed the boxing. fig 1h - I don’t know what this is nor what the labels refer to. it is not discussed anywhere in the main text.

- Original Fig. 1h has been removed and substituted with Figures 1g and 1j. These figures are now referenced in the paragraph: “SEB3R step 2.” delta f over f is also know as percent change. but there can be problems demonstrating effects this way, as mentioned above. the authors depict it as a linearly continuous value when it is not. I strongly recommend using a contrast ratio instead. also, delta f over f is a weighted term, already in extensive use for calcium imaging. consider another term?

- We have changed delta F over F in Delta SHAM over SHAM, for clarity. We fixed statistical analysis (also addressed in response to main point n.2). figure 3 - fig 3 is very difficult to interpret. x axes need labels on all of the plots, it took me 10 minutes to find them in j. I’d also suggest that the x axis shouldn’t be by body part, but instead, be the x position corresponding to the y position on the y axis. if the body part is important, different marker shapes could be used and designated in the legend. The heat map has no scale bar, we’re just told that green is some distance from p=0.5.

- We acknowledge reviewer’s concerns about figure 3. To make it easier to the reader, the figure was eliminated and relative parts readapted. The graphs depicting a schematic view of the postures are now recapitulated in fig.2, along with a snapshot of the actual body-posture extracted from the original videos. The graphs showing changes in Δsham/sham are now merged with the heatmap showing BMs% changes compared to the open field session. Accordingly, the two results paragraphs relative to these figures were combined in the paragraph titled “BMs identified using SEB3R discriminate stimulus-driven changes in mouse behavior”. As for the graphic of the posture-plots, we believe it is easier for the reader to understand by having the body-points equally spaced. Moreover, we would advise against using different markers for each body part, which would include additional visual complexity. If the reviewer and the editor believe that these changes are necessary to accept the manuscript it is no problem for us to change it accordingly. fig 4 - there are no units on time spent evading and again, just generally cluttered and hard to find my way.

- We added the scale units for the chart as suggested, removed the boxing and fixed the image quality accordingly. Also, we changed the shape of the first chart to make it more visible. fig 5 - qualitatively, the two images are just brighter/dimmer overall. perhaps I’m missing something. adding the points/regression for intensity in the 5a plots for LA might help?

- We added the correlation data for the Lateral Amygdala as supplementary figure 7 to support our statement in the main text. Thanks to the Reviewer’s comment, we also realized that the original version of image 6B, mouse 062 was erroneously too dim. We now present the correct version of the image, with a luminosity comparable to that of mouse 213 and obtained with the same acquisition parameters as those used for mouse 213. I have no idea what this is saying “To probe for neural activation, we used immunofluorescent labeling of ARC, a neuroplastic protein involved in stress response in the amygdala (Dirven et al, 2022), consistently with previous studies showing increased expression of immediate-early genes in response to fear (Campeau et al, 1991) and whisker stimulation (Chelini et al, 2019).”

- We apologize for the confusion; the second half of this period was part of a preliminary version of the manuscript and escaped our attention during the final edits. We removed it and changed the sentence to make it more understandable: “To probe for neural activation, we used immunofluorescent labeling of ARC, a protein coded by an immediate early gene whose expression in the amygdala is activated by stressful stimuli (Dirven et al, 2022).”

Minor: on page 4, DLC is cited but DLC-3d is used

- Where appropriate, we always use the same two references for DeepLabCut-3D (Nath et al, 2019. Mathis et al., 2018) as kindly requested on the DeepLabCut GitHub page for using their code. However, we realized that the date of the first reference was mistaken and fixed it accordingly. “marked and significant” on page 8 and other places - what is marked in this case, and how is it different from significant?

- We removed the word ‘marked’ that was only generating confusion. please proofread. there are several places where the subject/ verb count are off (“correlations was observed”) , missing ‘the’, etc - Done there are already several acronyms in the paper. could BC become categories or behavioral categories?

- Removed the acronym as suggested Information that could be helpful in revising the manuscript: SLEAP is designed to work for black and white video and in general provides better pose estimation than DLC or DLC-3D, which uses hue and therefore struggles with black and white. Other code, like anipose and pose3D can provide 3d position. Is DLC3d necessary for your pipeline? How would it work with data from other tools for pose estimation?

- This pipeline is conceived to specifically use DeepLabCut 3D outputs files as a starting point, as repeatedly specified during the manuscript. We have not tested it with other tracking systems. While we acknowledge this limitation, we think it does not invalidate SEB3R usefulness.

Reviewer 2

The goal of the WNt3R technique provided in the manuscript is to replace manual scoring of behavioral quantification, which has been shown to previously be problematic due to its subjective and time-consuming nature. This is a very straightforward study showing mostly proof-of-concept of a new toolbox that can be used with output from deeplabcut. The authors provide nice comparison of manual scoring with their automated method, and show proof-of-concept of a pipeline for how to utilize output from DLC-3D, which is also a missing link in many experimental efforts recently (i.e. DLC is used in a study and applied to video data, but there is an open range of how to utilize/analyze and what to do with DLC output). This study offers a method for analyzing and quantifying behavioral posture into certain behavioral clusters, based on DLC-3D output and k-means clustering. From this, the authors discover several differences across tested groups, for example, in posture based solely on the presence of a novel stimulus, regardless of if that stimulus is tactile / touches the animal. They also found differences in animal posture when comparing mild to heavy habituation to the whisker stimulus. The authors then correlated manual scoring behavioral categories with their cluster-defined behavioral modules. Finally, they used their behavioral clustering pipeline to attempt to correlate individual behavior clusters with stress related activity in the amygdala. Overall, the study advances the field by providing an automated pipeline for analysis of the whisker nuisance test. I have a few points that should be addressed by the authors.

Major:

1. Several details should be included to provide readers with step-by-step walk-through of the pipeline. First, a schematic or diagram of the automation pipeline should be included, to provide readers with an idea of the analysis. This is attempted in Figure 1F, but is quite vague. An updated version of this schematic should describe that these are all being performed by the pipeline itself and not manually by the experimenter. Relatedly, a screengrab or description of what Matlab is producing at each stage/level of the pipeline should be included. There’s some nice detail in the supplement on the instruction manual for the toolbox that can be included in the methods as well (such as camera resolution, etc).

- The methods section now includes a step-by-step guide to the pipeline. Additionally, we added some details to the image (in Figure 1) that shows the workflow of the pipeline and provided a graphical abstract to help readers navigate the paper. As suggested, we included step-by-step screengrabs of the MATLAB outputs as extended figures 1-6. (Also addressed in response to main point n.3)

2. Additionally, data formats are not discussed at all, nor are specifics of the types of cameras (frame rate, etc) needed for these analyses. Some of this may be able to be pulled from the supplement, but should really also belong in the main text. Also, are there specifics to the DLC-3D model training that need to be used? It would be helpful to have a set of parameters provided that enable step-by-step processing for others to follow to a T.

- We have included some details where appropriate, but there are not major requirements beyond the use of DeepLabCut. The lack of major technical requirements is, in our opinion, one of the main advantages of SEB3R, as highlighted in the discussion.

3. How much can this pipeline be extrapolated and utilized for other behavioral movement tasks? Or is this only intended and envisioned for this one task? If so, it seems to really reduce the usability of the tool to a very specific group of researchers, but one can envision many other uses for this similar type of pipeline. For example, in a head-fixed mouse, could a similar whisker manipulation task be performed and the data analyzed in a similar way? Can this in theory be used in other stress-related studies where animals exhibit freezing behavior? Can this be used in other species, such as freely-moving rats, in a similar manner? These should all be demonstrated, if possible, or at least discussed in the manuscript.

- We have substantially edited the manuscript to make SEB3R more broadly available for additional uses; please see response to main point n.1

- The tool is meant to track freely moving mice and would not work in head-fixed.

- Speculatively, the system could work in rats, but we are in no position to directly test it, so we avoided mentioning it in the manuscript.

Minor:

1. There is a bit of confusion at times on describing the BC (behavior categories) and BM (behavior modules); as I understand the BC is operationally defined by the experimenter, but the BM# is the specific post-DLC/post-kmeans clustered groups. This is described nicely when correlating the manual BC to BM, as in figure 4, but this should be made clear in the introduction and methods of the specific definition of these. This seems to be the major component of the study to be able to compare with previous studies that used manual behavioral scoring and should be emphasized further.

- Removed the acronym as suggested

2. How does this method compare with other post-DLC-3D analysis methods out there? Do other methods exist to take DLC-3D output and make behavioral analyses from there? There should be a point in the discussion that compares against other post-pose estimation video tracking methods.

- We have discussed advantages and limitations of SEB3R in the discussion

3. Additional typos/notes:

- Page 3, paragraph 2: replace “constrains” with “constraints” (noun)

- Page 5, first line: replace “consisted in” with “consisted of”

- Page 5, first paragraph: replace “Fearful behaviors were divided in” with “divided into”

- Page 5 second paragraph: replace videocameras with “video cameras”

- Page 5 second paragraph: there is an extra “)”

- Page 5 second paragraph: replace “eachother” with “each other” ; “respect” can be deleted in the same sentence

- Page 5 second paragraph: For clarity, replace “at every given animal” with “after” or “with”

- Fig 1f - keep the abbreviations consistent; WiNT3R should be “WNt3R” like in the rest of the text

- Page 6 - replace git-hub with GitHub

- Figure 1 (i) identify the abbreviations of No,E,N,mB,IB,T in the figure caption

- All these additional issues were fixed accordingly, where still relevant
