# Supplementary figures and images for: Automated Segmentation of the Mouse Body Language to Study Stimulus-Evoked Emotional Behaviors
Source: eNeuro. 2023 Sep 8;10(9):ENEURO.0514-22.2023. doi: 10.1523/ENEURO.0514-22.2023 (PMC10496135; doi:10.1523/ENEURO.0514-22.2023)

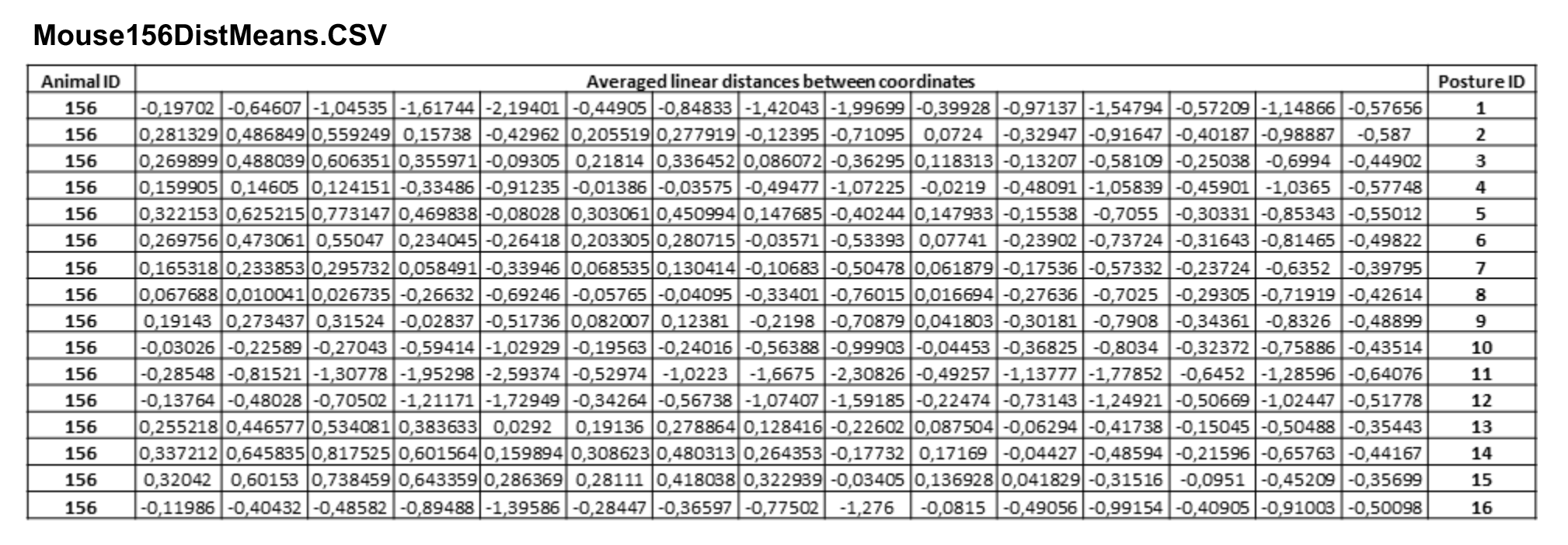

Supplement: Extended Data Figure 1-1 — Example of the output file MouseIDDistMean.CSV provided at the end of step 1 and required to run step 2 for one representative mouse (animal number 156). Download Figure 1-1, TIF file. [file enu-eN-MNT-0514-22-s22.tif]

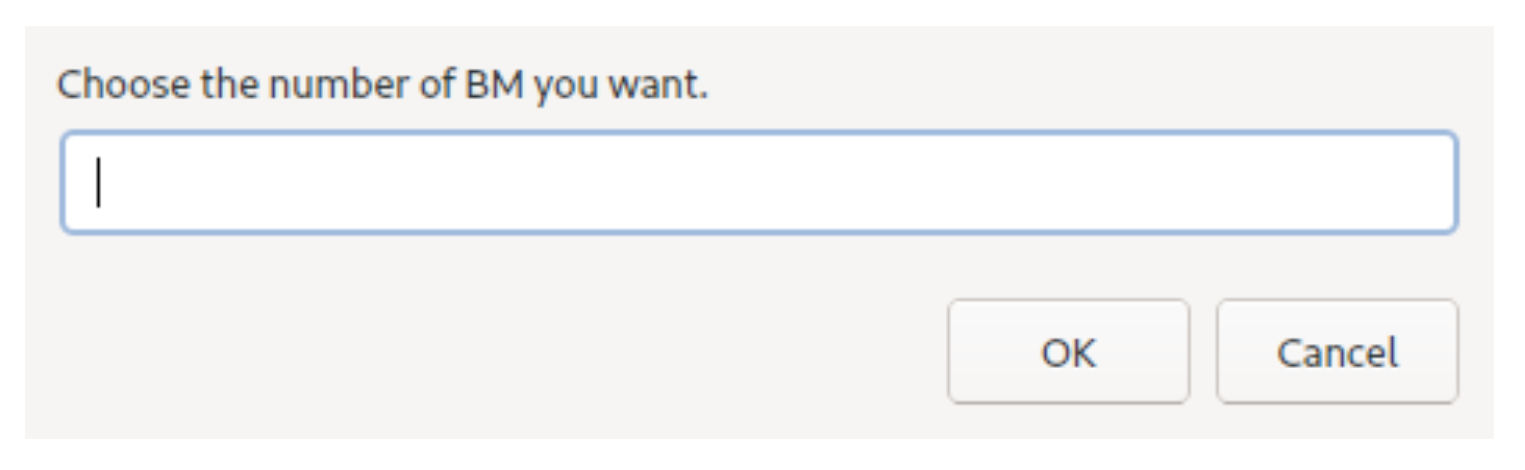

Supplement: Extended Data Figure 1-2 — Pop-up window asking the user to indicate the final number of k-means required to run the step 2. The indicated k-mean will correspond to the final number of BMs. Download Figure 1-2, TIF file. [file enu-eN-MNT-0514-22-s23.tif]

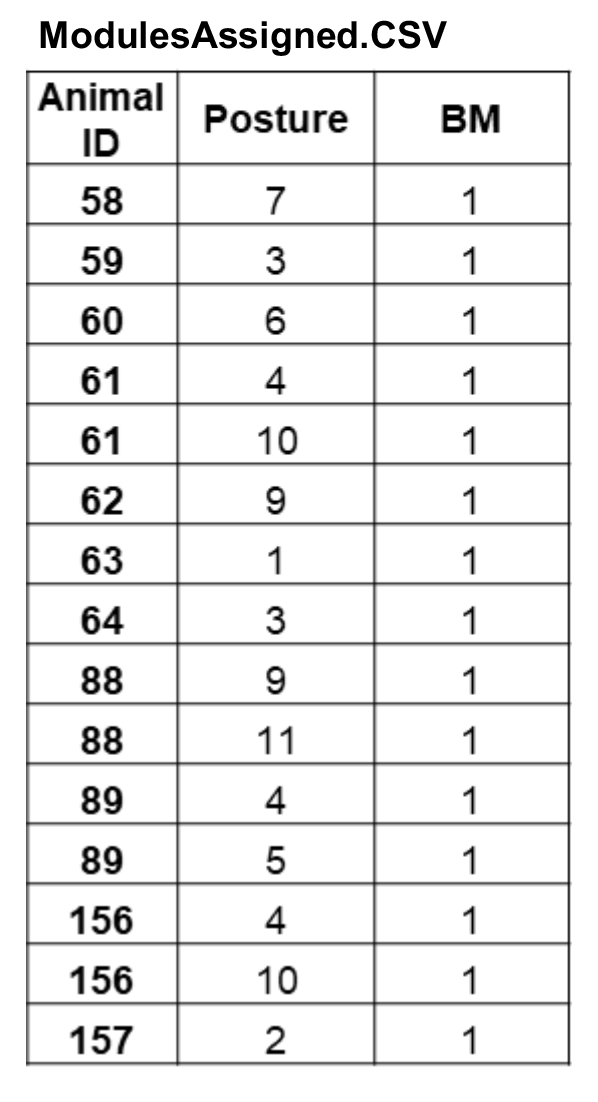

Supplement: Extended Data Figure 1-3 — Example of the output file ModulesAssigned.CSV provided at the end of step 2 and required to run step 3. Download Figure 1-3, TIF file. [file enu-eN-MNT-0514-22-s24.tif]

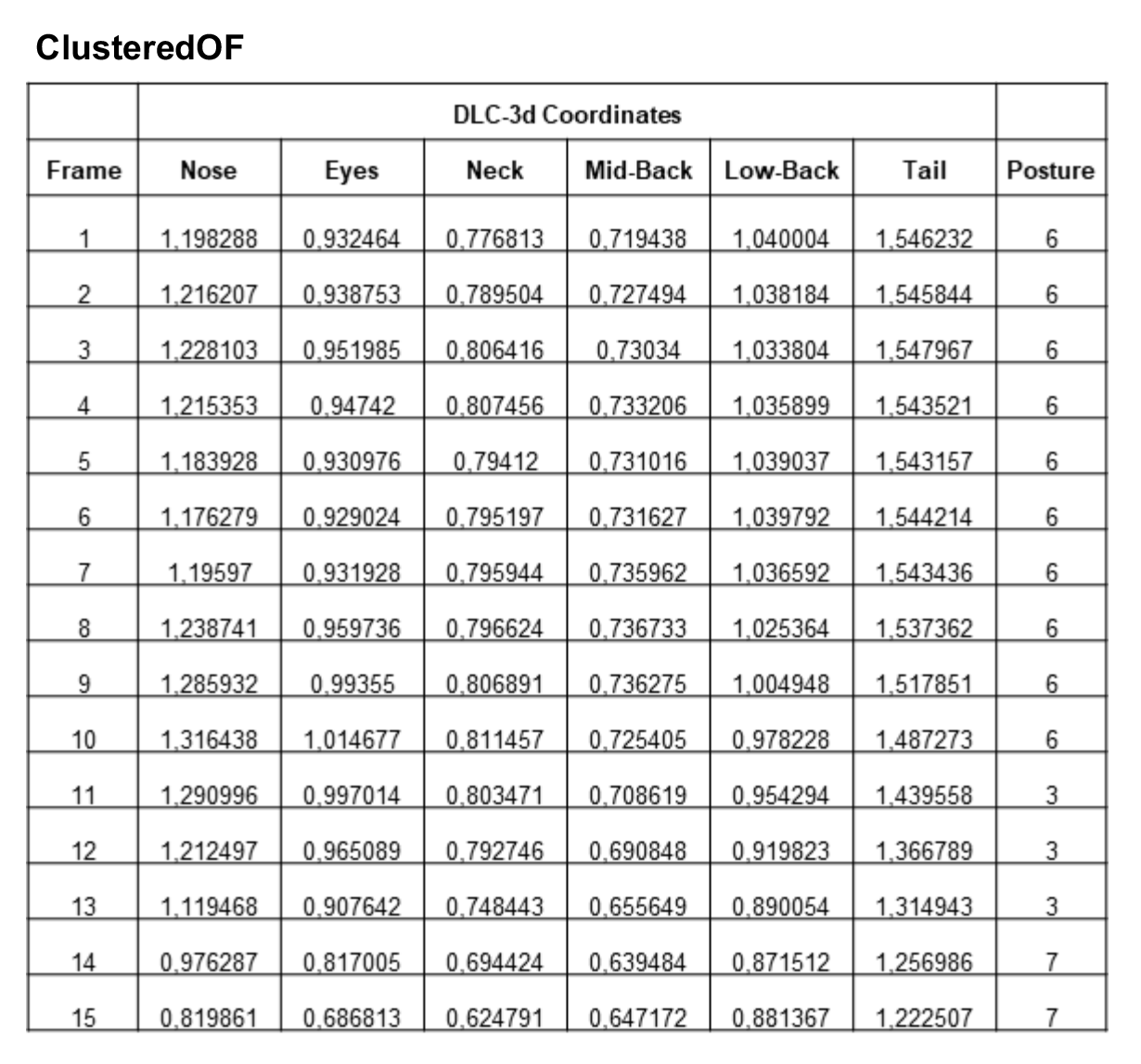

Supplement: Extended Data Figure 1-4 — Example of the output file Clustered.CSV provided at the end of step 1 and required to run step 3, for a representative mouse. OF in the file name indicates that this file belongs to an open field session. Download Figure 1-4, TIF file. [file enu-eN-MNT-0514-22-s25.tif]

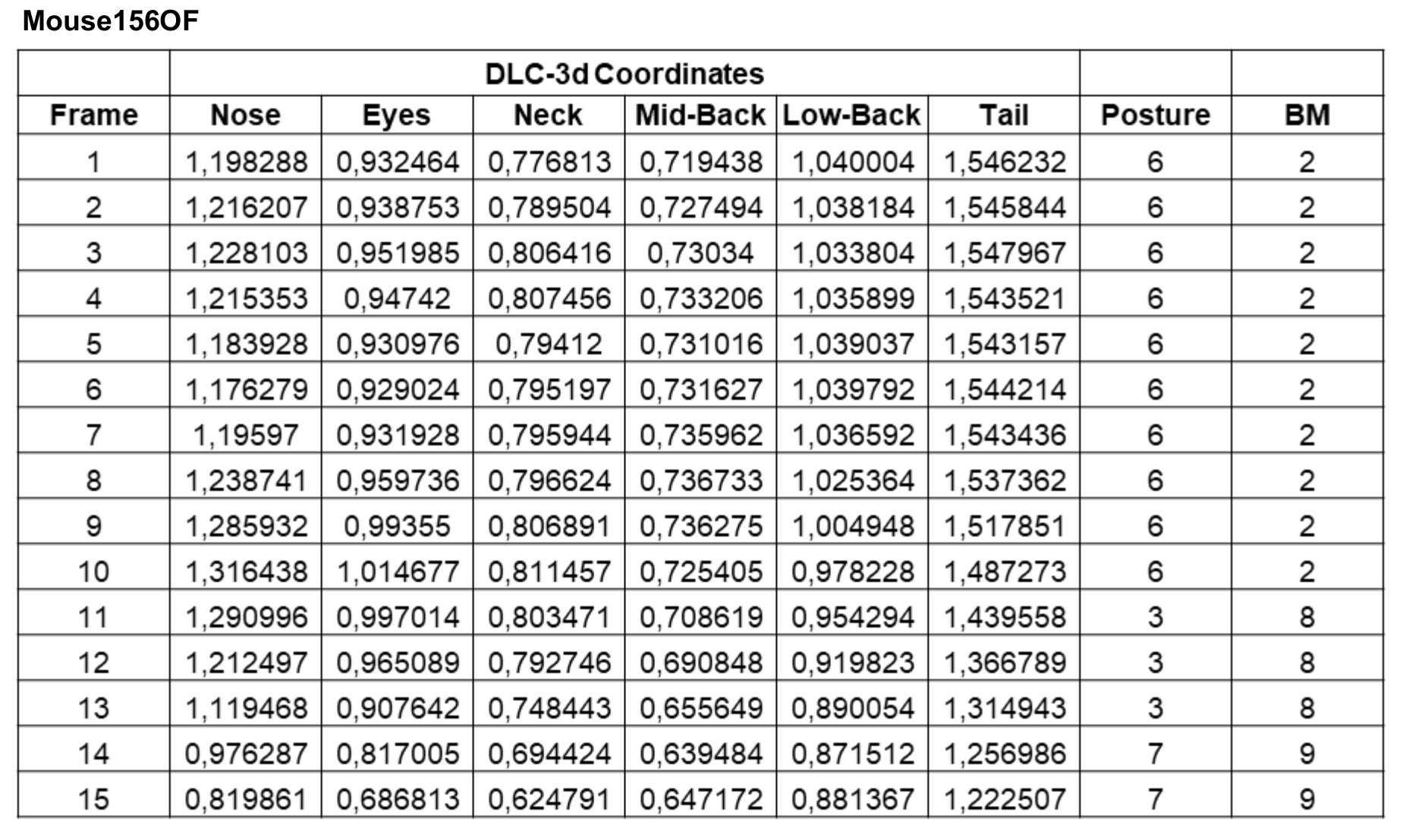

Supplement: Extended Data Figure 1-5 — Example of the final output of the pipeline provided for a single mouse (mouse no. 156). OF in the file name indicates that this file belongs to an open field session. Download Figure 1-5, TIF file. [file enu-eN-MNT-0514-22-s26.tif]

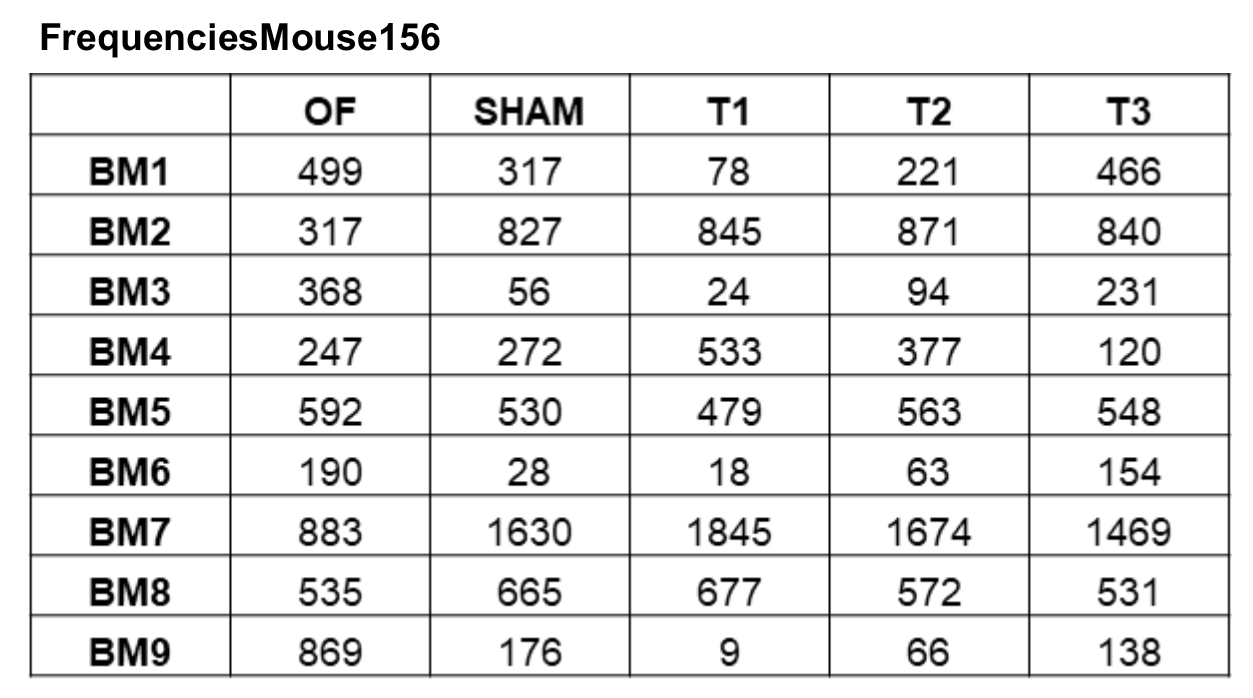

Supplement: Extended Data Figure 1-6 — Example of the final output of the pipeline recapitulating the total number of frames assigned to each independent BM for a representative mouse (mouse no. 156) across the five consecutive sessions of the WN. Download Figure 1-6, TIF file. [file enu-eN-MNT-0514-22-s27.tif]

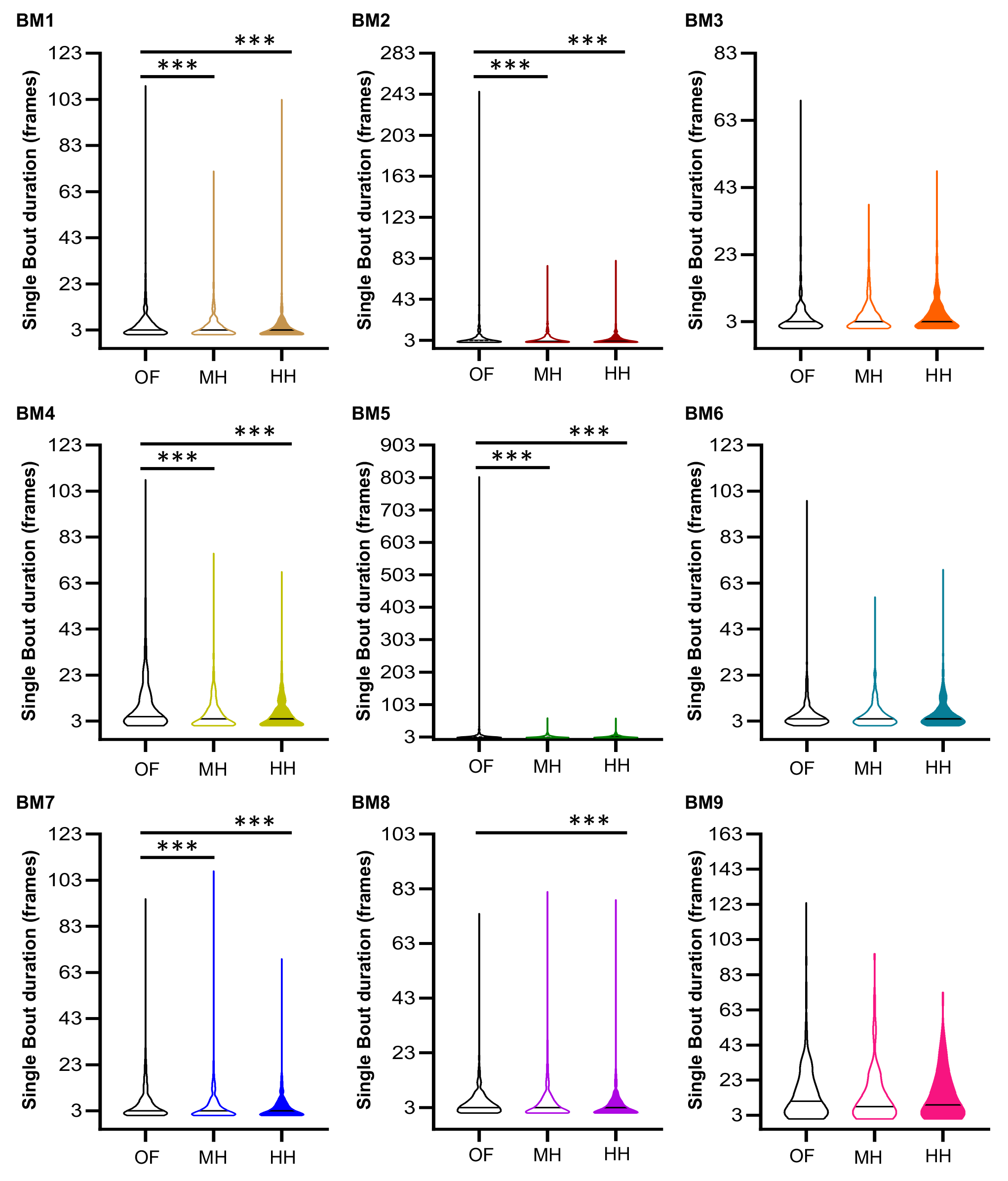

Supplement: Extended Data Figure 3-1 — Violin plots depicting the variability in the duration of behavioral bouts sorted by BMs. Note how exclusively curiosity-driven BMs (3-6-9) do not change between experimental conditions. The color code for each BM is the same used in Figures 2–5 and Extended Data Figure 4-1. Download Figure 3-1, TIF file. [file enu-eN-MNT-0514-22-s28.tif]

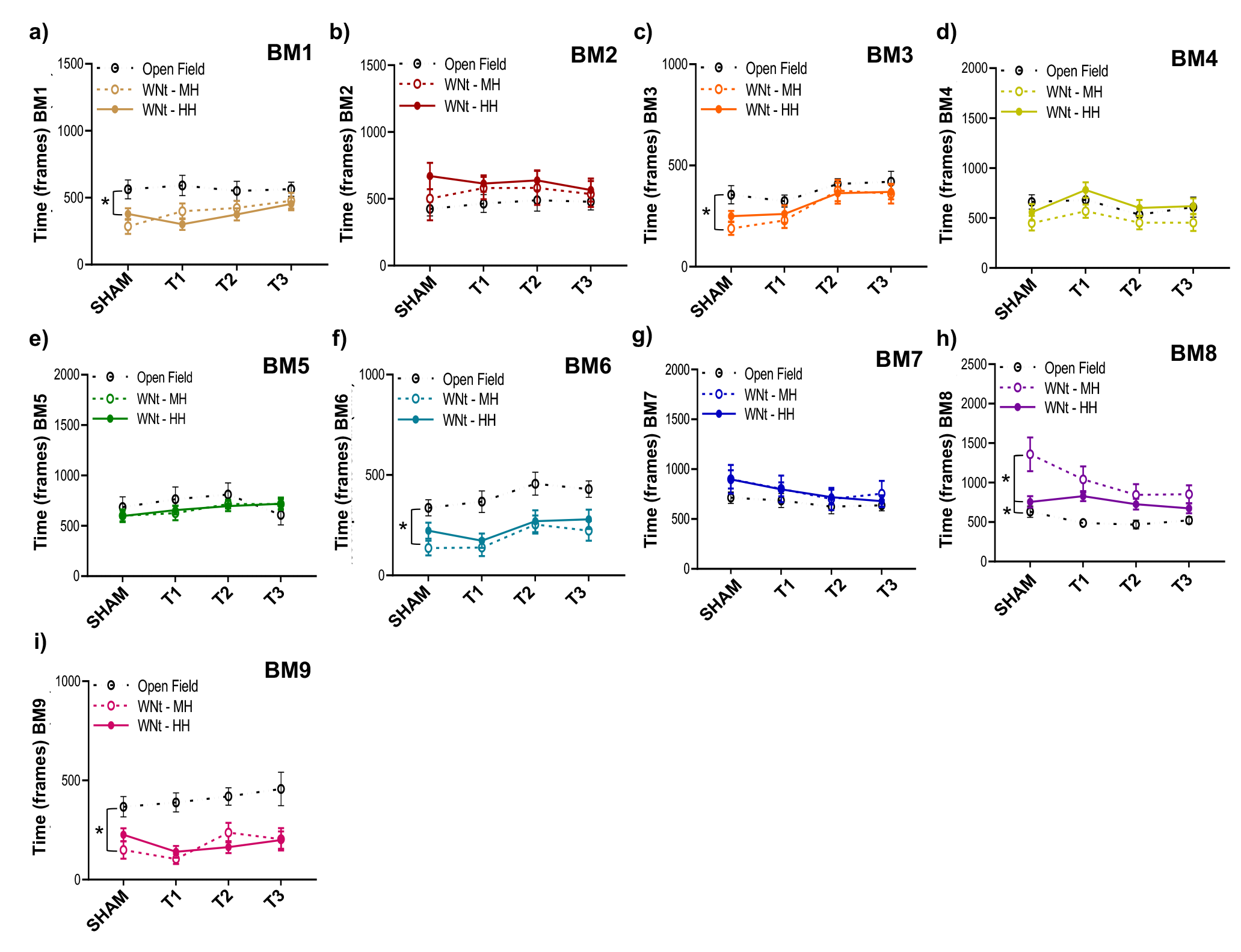

Supplement: Extended Data Figure 4-14 — a–i, Time spent in BMs for the three experimental conditions. Note how, because of the differences in experimental settings, the absolute time spent in BMs during the SHAM session changes across experimental groups, rendering the interpretation of stimulus-driven behavior inconsistent. This discrepancy can be overcome by using the SHAM session as a baseline reference to normalize and express the relative time spent in BMs as percentage change compared to SHAM in response to the whisker stimulation. Download Figure 4-14, TIFF file. [file enu-eN-MNT-0514-22-s29.tif]

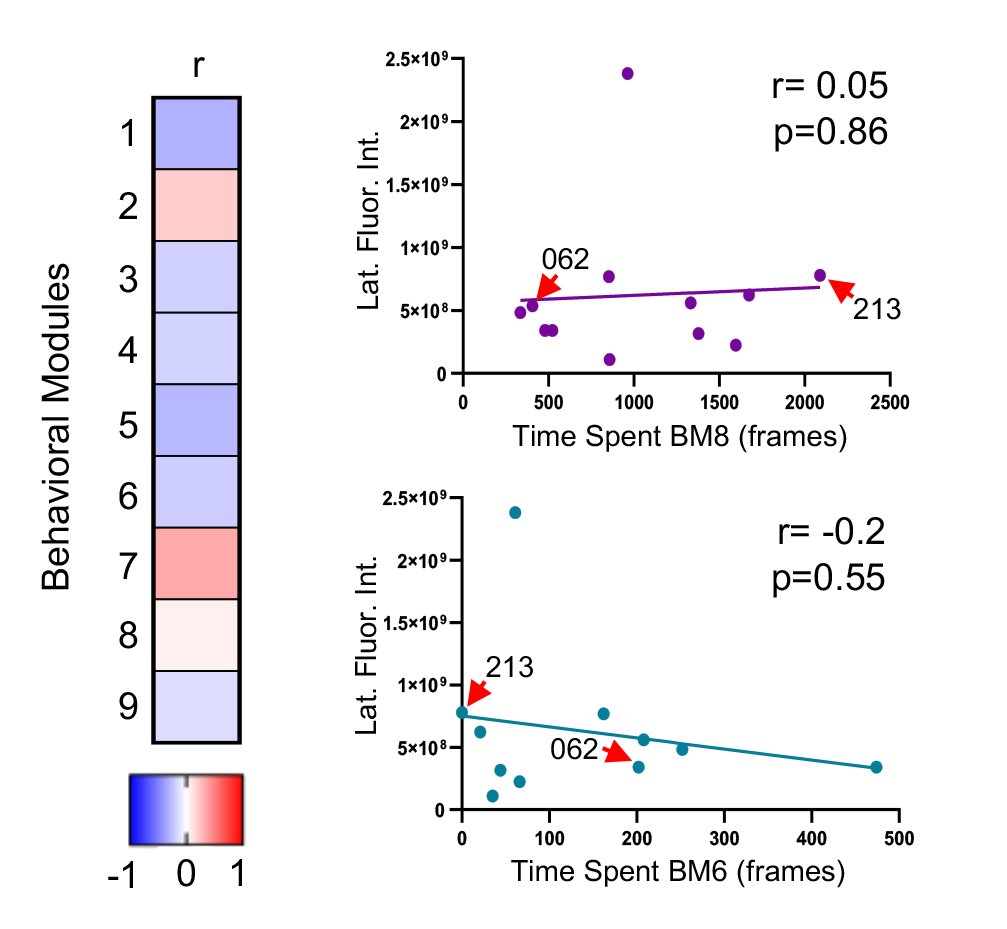

Supplement: Extended Data Figure 6-1 — Arc protein expression in lateral amygdala does not show correlation with BMs. Download Figure 6-1, TIF file. [file enu-eN-MNT-0514-22-s30.tif]
